# A Transcription Elongation Factor That Links Signals from the Reproductive System to Lifespan Extension in *Caenorhabditis elegans*

**DOI:** 10.1371/journal.pgen.1000639

**Published:** 2009-09-11

**Authors:** Arjumand Ghazi, Sivan Henis-Korenblit, Cynthia Kenyon

**Affiliations:** 1 Department of Biochemistry and Biophysics, University of California San Francisco, San Francisco, California, United States of America; Stanford University Medical Center, United States of America

## Abstract

In *Caenorhabditis elegans* and *Drosophila melanogaster*, the aging of the soma is influenced by the germline. When germline-stem cells are removed, aging slows and lifespan is increased. The mechanism by which somatic tissues respond to loss of the germline is not well-understood. Surprisingly, we have found that a predicted transcription elongation factor, TCER-1, plays a key role in this process. TCER-1 is required for loss of the germ cells to increase *C. elegans'* lifespan, and it acts as a regulatory switch in the pathway. When the germ cells are removed, the levels of TCER-1 rise in somatic tissues. This increase is sufficient to trigger key downstream events, as overexpression of *tcer-1* extends the lifespan of normal animals that have an intact reproductive system. Our findings suggest that TCER-1 extends lifespan by promoting the expression of a set of genes regulated by the conserved, life-extending transcription factor DAF-16/FOXO. Interestingly, TCER-1 is not required for DAF-16/FOXO to extend lifespan in animals with reduced insulin/IGF-1 signaling. Thus, TCER-1 specifically links the activity of a broadly deployed transcription factor, DAF-16/FOXO, to longevity signals from reproductive tissues.

## Author Summary

The reproductive status and longevity of animals are strongly interlinked. Increasing age influences the reproductive capacities of most animals. However, little is known about how reproductive status might affect lifespan. Experiments in worms and flies have shown that removing cells that give rise to gametes, the “germ cells”, makes them live longer. We know very little about the genes and molecules that are involved in this process. In this study, we have identified a gene called *tcer-1* that promotes the longevity of the roundworm *Caenorhabditis elegans* when its germ cells are removed. The gene *tcer-1* codes for a protein, TCER-1, that is predicted to function as a “transcription elongation factor” (it allows the completion of RNA synthesis during the process of gene expression). Our experiments imply that when the germ cells of worms are removed, TCER-1 collaborates with a transcription factor called DAF-16/FOXO to express genes that contribute to increased longevity. DAF-16/FOXO can extend lifespan in response to other physiological cues besides loss of germ cells. However, TCER-1 specifically helps this widely used longevity protein to respond to signals that reflect the reproductive status. Counterparts of DAF-16/FOXO are known to control aging in other organisms, including humans, so the identification of TCER-1 may lead to a better understanding of the relationship between reproduction and aging in other species, too.

## Introduction

When the germline of *C. elegans* is removed, either by laser ablation or by mutations that block germline-stem cell proliferation, the animals live approximately 60% longer than normal [1],[2]. This longevity is not a simple consequence of sterility, as removing the entire gonad (the germ cells as well as the somatic reproductive tissues) does not extend lifespan [1]. These and other findings suggest that both the germline cells and the somatic reproductive tissues influence lifespan [3],[4]. A link between reproductive tissues and aging may be widespread in nature, as removing germline-stem cells during adulthood increases the lifespan of flies [5], as well as worms, and transplanting ovaries of young mice into older animals can increase lifespan as well [6].

The mechanism by which loss of the germline extends lifespan is not well understood. At least two transcription factors, the FOXO-family transcription factor DAF-16/FOXO [7]–[9] and the nuclear hormone receptor DAF-12 [10], are required for germ-cell loss to extend lifespan in *C. elegans* [1]. When germ cells are removed, DAF-16/FOXO accumulates in nuclei, primarily in the animal's intestine [11]. The intestine, which functions as the worm's entire endoderm, including its fat tissue, appears to play a key role in this pathway, as expressing DAF-16/FOXO only in the intestine completely restores the lifespan extension of germline-defective *daf-16(−)* animals [12]. DAF-16/FOXO nuclear localization requires an ankyrin-repeat, intestinal protein called KRI-1 [13]. In addition, full DAF-16/FOXO nuclear localization requires DAF-12/NHR [10],[13], as well as DAF-9, a cytochrome P450 protein that synthesizes a DAF-12/NHR ligand [13]–[15].

DAF-16/FOXO is particularly interesting because of its evolutionarily-conserved role in another lifespan regulatory pathway, the insulin/IGF-1 endocrine pathway [16]–[18]. In wild-type worms, the insulin/IGF-1 receptor DAF-2 activates a series of conserved kinases that ultimately phosphorylate and inactivate DAF-16/FOXO [19]–[21]. When insulin/IGF-1 signaling is inhibited, DAF-16/FOXO accumulates in nuclei, where it regulates the transcription of downstream antioxidant, chaperone, innate-immunity, and metabolic genes that more directly affect lifespan [8], [9], [11], [22]–[25]. Insulin/IGF-1 signaling and FOXO proteins influence lifespan in worms, flies and mice [16],[26],[27]. The pathway appears to affect human longevity as well, as variants in the human Foxo3a gene have been linked to longevity in several human populations [28]–[32], and functionally significant IGF-1 receptor mutations are overrepresented in populations of centenarians [33].

In an effort to better understand how reproductive cues trigger lifespan extension, we carried out a genetic screen for RNAi clones that prevent germline loss from extending lifespan. Surprisingly, in our screen we identified a gene, *tcer-1*, that encodes the homolog of the human transcription elongation factor TCERG1. Loss of *tcer-1* in *C. elegans* sharply curtails the lifespan extension produced by removal of the germline, but has little or no effect on wild-type lifespan. Thus, TCER-1 is not likely to be a general component of the transcriptional machinery; instead, it has a much more specific function in the animal.

We find that TCER-1 plays a key regulatory role in transducing signals from the reproductive system to the somatic tissues. When the germline is removed, TCER-1 levels rise in the intestine. This up-regulation is functionally significant because overexpressing *tcer-1* in normal, fertile animals bypasses the requirement for germ-cell loss and extends lifespan. Our findings indicate that the role of *tcer-1* in the reproductive longevity pathway is to promote the transcription of a subset of DAF-16/FOXO-target genes that are up-regulated upon germline removal. Thus, TCER-1 appears to act in association with DAF-16/FOXO to extend lifespan.

Interestingly, *tcer-1* is not invariably required for DAF-16/FOXO transcriptional activity, as it is not needed for DAF-16/FOXO to up-regulate its target genes or to extend lifespan in *daf-2* insulin/IGF-1 receptor mutants. At least some of the genes that are up-regulated by DAF-16/FOXO in a *tcer-1*-dependent fashion in long-lived, germline-defective animals do not require *tcer-1* for their expression in long-lived insulin/IGF-1 mutants. Thus, this transcription elongation factor appears to link germline loss to a precise DAF-16/FOXO-dependent transcriptional program.

## Results

### *tcer-1* is required for the longevity associated with germline ablation

**Figure 1:**
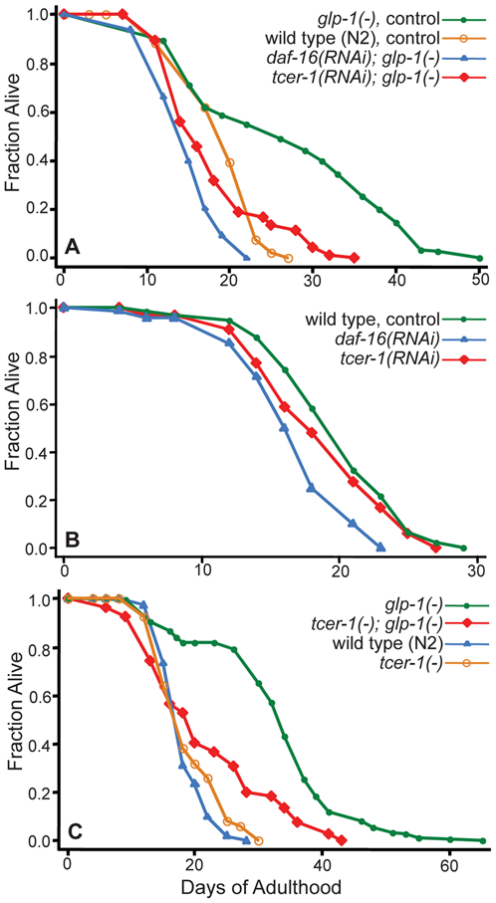
Reduction of *tcer-1* function suppresses the lifespan extension produced by germline ablation. (A) Effect of *tcer-1* RNAi on the lifespan of germline-defective mutants. *glp-1* (germline-defective) mutants grown on bacteria containing control empty vector (m = 27.0±1.1, n = 112/114), *daf-16* RNAi bacteria (m = 15.2±0.2, n = 110/113, *P* vs. control<0.0001), and *tcer-1* RNAi bacteria (m = 18.1±0.4, n = 96/114, *P* vs. control<0.0001). Wild-type (N2) worms grown on bacteria containing control empty vector shown for comparison (m = 19.5±0.3, n = 81/99, *P* vs. *glp-1(−)*<0.0001). (B) Effect of *tcer-1* RNAi on the lifespan of wild-type (N2) worms. N2 worms grown on bacteria containing control empty vector (m = 19.9±0.6, n = 49/72), *daf-16* RNAi bacteria (m = 16.7±0.5, n = 62/72, *P* vs. control<0.0001), and *tcer-1* RNAi bacteria (m = 18.7±0.5, n = 66/72, *P* vs. control = 0.2, *P* vs. *daf-16* RNAi = 0.002). (C) Effect of the *tcer-1(tm1452)* mutation on the lifespan of *glp-1* mutants and wild-type worms. *glp-1(−)*: m = 32.9±0.3, n = 233/258; *tcer-1(−)*; *glp-1(−)*: m = 21.8±0.5, n = 77/115, *P* vs. *glp-1(−)*<0.0001; wild type (N2): m = 18.9±0.5, n = 60/121; *tcer-1(−)*: m = 19.0±0.4, n = 96/110, *P* vs. N2 = 0.84. Additional repeats of these experiments are shown in Table S1.

We identified *tcer-1* in a Chromosome II RNAi screen for genes required to extend the lifespan of germline-depleted *glp-1* mutants (see Materials and Methods). When grown at the non-permissive temperature (25°C), temperature-sensitive *glp-1* mutants are sterile because the germline-stem cells fail to proliferate [34]. These mutants recapitulate the lifespan extension of animals whose germline-precursor cells have been eliminated by laser ablation [2]. Since lifespan can be shortened by conditions that simply compromise the animals' health, we focused on RNAi clones that suppressed the extended lifespan of *glp-1* mutants but had little or no effect on the lifespan of wild-type animals. From this screen, we identified a clone of the gene *tcer-1* (*ZK1127.9*), which encodes the *C. elegans* homolog of a human transcription elongation factor, TCERG1 (also known as CA150) [35]–[37]. *tcer-1* RNAi strongly suppressed the extended lifespan of *glp-1* mutants, but had no effect on the lifespan of wild-type animals (Figure 1A and 1B; Table S1A). Wild-type animals grown on bacteria expressing *tcer-1* dsRNA appeared normal and healthy, had regular developmental rates and displayed normal reproduction.

We obtained a *tcer-1* mutant, *tcer-1(tm1452)*, from the *C. elegans* National Bioresource Project, Japan. The *tm1452* mutation is a 10 bp insertion coupled to a 392 bp deletion that eliminates parts of the WW domains of the protein and is likely to reduce gene function. We constructed a *tcer-1*; *glp-1* double mutant and found that it had a much shorter lifespan than did *glp-1* mutants; for example, in one experiment the 74% extension in lifespan produced by the *glp-1* mutation was reduced by *tcer-1(tm1452)* to 15% (Figure 1C, Table S1B). There was no effect on the lifespan of wild-type worms (Figure 1C, Table S1C). This finding is significant, as it indicates that *tcer-1* is unlikely to play a general role in transcription elongation, but instead has a more specific function in the animal. *tcer-1* mutants displayed a delay in developmental timing, and a modest reduction in brood size that was not elicited by *tcer-1* RNAi treatment (data not shown).

### Germline loss elevates TCER-1 levels in the intestine

**Figure 2:**
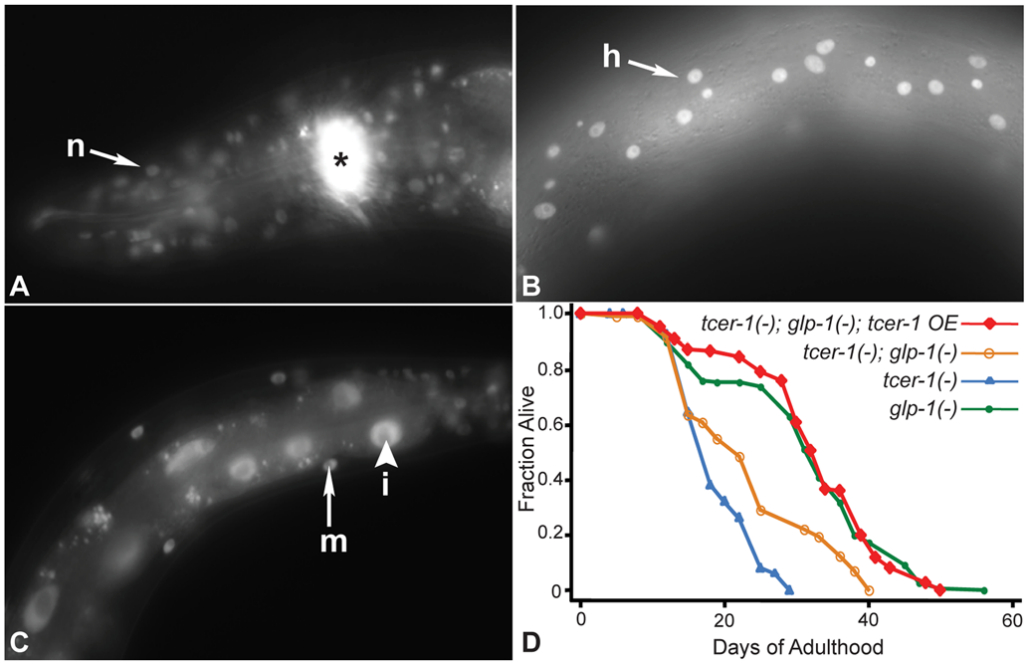
A TCER-1::GFP fusion protein is widely expressed and rescues the shortened lifespan of *tcer-1*; *glp-1* mutants. (A–C) *tcer-1* expression in adult somatic tissues. TCER-1::GFP is expressed in nuclei of adult neurons (‘n’, head neurons shown by arrow in (A)), hypodermis (‘h’, (B)), muscles (‘m’, arrow in (C)), and intestinal cells (‘i’, arrowhead in (C)). The asterisk in (A) indicates expression of the co-injection marker, *Podr-1::RFP*, in olfactory neurons. (D) Rescue of the shortened lifespan of *tcer-1(−)*; *glp-1(−)* mutant by the TCER-1::GFP fusion protein (*tcer-1 OE*). *glp-1(−)*: m = 31.1±0.7, n = 204/241; *tcer-1(−)*; *glp-1(−)*: m = 23.4±0.5, n = 81/119, *P* vs. *glp-1(−)*<0.0001; *tcer-1(−)*; *glp-1(−)*; *tcer-1 OE*: m = 32.4±0.3, n = 111/116, *P* vs. *glp-1(−)* = 0.5, *P* vs. *tcer-1(−)*; *glp-1(−)*<0.0001; *tcer-1(−)*: m = 19.1±0.2, n = 50/64. See Table S1. C for additional trials of this experiment.

The observation that reducing *tcer-1* activity had only mild effects on wild type but almost completely prevented germline loss from extending lifespan suggested that *tcer-1* might play a regulatory role in this pathway. If so, it seemed possible that its level or location in the animal might change upon germline removal. To investigate this possibility, we constructed transgenic animals expressing a TCER-1::GFP fusion protein under the control of the endogenous *tcer-1* promoter (see Materials and Methods). In the wild type, TCER-1::GFP was visible at all stages of embryonic and larval development (data not shown). In adults, we observed strong nuclear localization of TCER-1::GFP in intestinal cells, many head and body neurons, muscle and hypodermal cells (Figure 2A–2C). In some intestinal cells, weak expression was also observed in the cytoplasm.

To test if this fusion protein was functional, we examined the ability of the construct to rescue the shortened lifespan of *tcer-1*; *glp-1* double mutants. Two independent lines expressing the TCER-1::GFP fusion protein completely rescued the lifespan of *tcer-1*; *glp-1* mutants in one trial (Figure 2D, Table S1C). In a second trial, the rescue was between 77–88% (Table S1C). Thus, TCER-1::GFP is a functional protein that likely reflects the endogenous expression of *tcer-1*. Importantly, these observations showed that a functional TCER-1 protein was present in intestinal nuclei in the adult, where DAF-16/FOXO has been shown to act to increase lifespan [11],[12].

**Figure 3:**
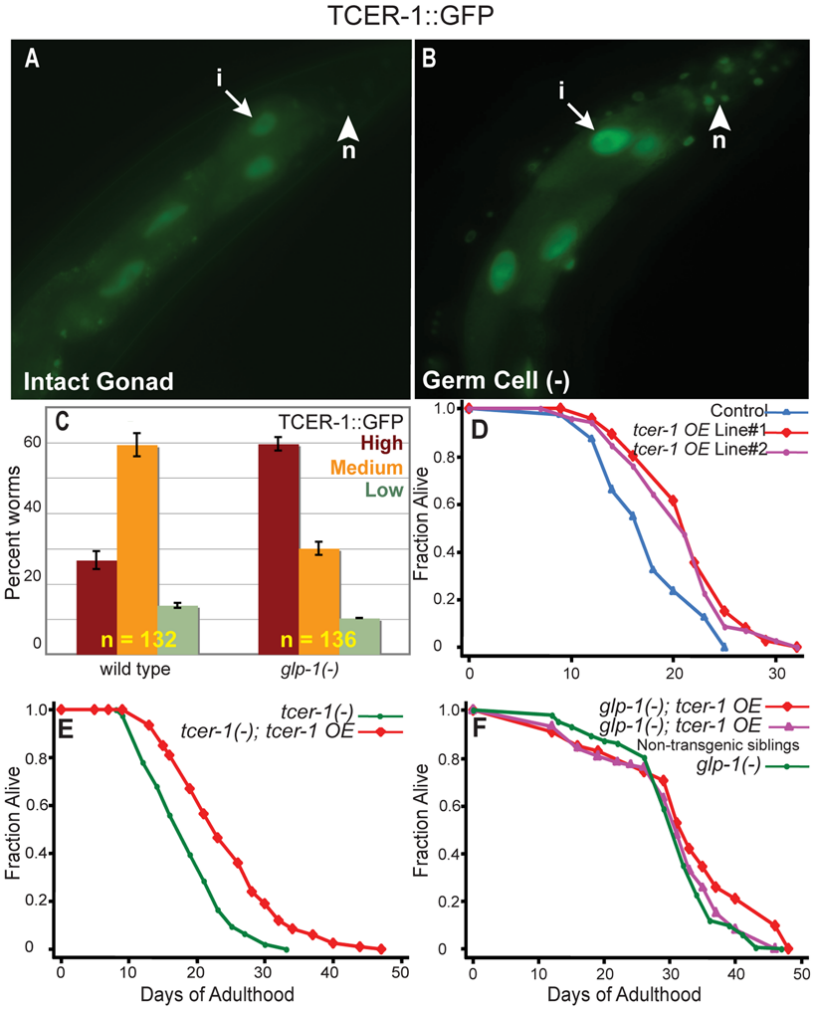
TCER-1 expression is elevated upon germline ablation and *tcer-1* overexpression increases the lifespan of wild-type animals. (A–C) Elevation of TCER-1::GFP levels following germline removal using laser ablation (A,B) or *glp-1* mutantions (C). (A,B) TCER-1::GFP expression in day 1 adults of control worms with intact gonads (A), and germ-cell precursor (Z2, Z3)-ablated worms (B). Increased expression is visible in intestinal nuclei (‘i’) and neurons (‘n’) (compare arrows and arrowheads, respectively, between (A) and (B)). (C) Bar graph showing increased TCER-1::GFP expression in sterile, long-lived *glp-1* mutants. Day 1 adults of each strain were classified into those with High (red bars), Medium (orange bars), and Low (green bars) GFP levels (focusing primarily on the intestine). ‘n’ signifies the total number of worms examined in two trials. Error bars represent the standard error of the mean. (D) Effect of *tcer-1* overexpression on the lifespan of intact, wild-type animals. Wild-type control carrying only the co-injection marker (*Podr-1::rfp*): m = 17.4±0.4, n = 81/89. TCER-1::GFP, *tcer-1 OverExpressing* strains (*tcer-1 OE*), Line #1: m = 21.2±0.4, n = 85/90, *P* vs. control<0.0001; Line #2: m = 21.6±0.6, n = 74/79, *P* vs. control 0.0002. (E) Effect of *tcer-1* overexpression on the lifespan of *tcer-1* mutants. *tcer-1(−)*: m = 18.6±0.4, n = 91/108; *tcer-1(−)*; *tcer-1 OE*: m = 24.6±0.8, n = 81/106, *P*<0.0001. *tcer-1* overexpression in a *tcer-1(−)* background produced a greater lifespan extension than *tcer-1* overexpression in *tcer-1(+)* background. For example, in the above experiment, *tcer-1* overexpression in a *tcer-1(−)* background produced a 33% lifespan extension as compared to 14% in a *tcer-1(+)* background (note the difference in X-axis scale between A and B). The reason for this difference is unclear, but it is similar to the trend exhibited by another longevity-promoting transcription factor, SKN-1 [68]. (F) Effect of *tcer-1* overexpression on the lifespan of *glp-1* mutants. *glp-1(−)*: 30.6±0.2, n = 102/104. *glp-1(−)*; *tcer-1 OE*: m = 30.4±0.3, n = 82/89, *P* vs. *glp-1(−)* 0.3. *glp-1(−)*; *tcer-1 OE* non-transgenic (wild-type) siblings: m = 30.6±0.2, n = 86/89, *P* vs. *glp-1(−)* 0.4, *P* vs. *glp-1(−)*; *tcer-1 OE* = 0.2. See Table S2 for results with additional transgenic lines and multiple repeats of above experiments.

Next, we examined the effect of germline ablation on the pattern of TCER-1::GFP expression. We found that eliminating the germline increased the level of TCER-1 in somatic cells (Figure 3A–3C). This finding raised the possibility that TCER-1 might have an important regulatory role in the animal's response to germline ablation. An independent microarray analysis designed to identify longevity genes whose expression is altered by germline ablation also found *tcer-1* amongst the top 10% of germline-regulated genes (M. McCormick and C. Kenyon, unpublished data). Thus, *tcer-1* is likely to be transcriptionally up-regulated following germline depletion. TCER-1 levels increased primarily in two tissues, the intestine and neurons, upon germline removal (Figure 3A and 3B). *tcer-1* RNAi suppressed longevity without affecting neuronal expression (Figure S1). Because neurons are relatively resistant to RNAi in *C. elegans*, this finding suggests that intestinal TCER-1 function, like intestinal DAF-16/FOXO, is likely to be particularly important for promoting longevity.

### *tcer-1* overexpression extends the lifespan of wild-type, fertile worms

The finding that loss of the germline elevated TCER-1 levels raised the possibility that TCER-1 might play a rate-limiting step in this longevity pathway. To test this, we asked whether overexpressing *tcer-1* in wild-type animals might bypass the requirement for germline loss and extend the lifespan of fertile animals. We found that *tcer-1* overexpression produced a modest but consistent increase in the lifespan of wild-type worms (average mean lifespan extension ∼15%; Figure 3D and 3E; Table S2A and S2B). We also found that, in keeping with its role in the reproductive pathway, *tcer-1* overexpression did not produce any further increase in the extended lifespans of worms whose germ cells had been eliminated either by the *glp-1* mutation (Figure 3F; Table S2C) or by laser ablation (Figure S2; Table S2C).

### *tcer-1* is required for expression of DAF-16/FOXO-target genes in response to germline removal

To understand why *tcer-1* was required for loss of the germ cells to extend lifespan, we asked whether *tcer-1* might impact DAF-16/FOXO function. DAF-16/FOXO undergoes nuclear accumulation primarily in intestinal cells following germline removal and we first explored the possibility that *tcer-1* was required for this step. However, we found that in *tcer-1(tm1452)* worms lacking a germline, DAF-16/FOXO accumulated normally in intestinal nuclei (Figure S3). Curiously, we noticed a small increase in the levels of DAF-16::GFP, largely in the cytoplasm of intestinal cells, in these animals (Figure S3; see Discussion).

Next, we examined the effect of *tcer-1(tm1452)* on DAF-16/FOXO's transcriptional output following germline loss. Many putative (direct or indirect) DAF-16/FOXO target genes have been identified [12], [22]–[25], but only a few, *sod-3*, *dod-8*, *gpd-2* and *nnt-1*, are known to be up-regulated by germline removal [3]. To increase the repertoire of such genes, we obtained available transgenic worm strains expressing GFP-tagged transcriptional reporters for additional putative DAF-16/FOXO targets [38] (see Table S3). Germline precursors of each of these strains were laser ablated, and the animals were examined as adults for changes in expression. We found several genes whose expression was sharply elevated upon germline removal, including *K07B1.4*, *T21D12.9*, *F52H3.5*, *aat-1* and *pssy-1* (see Table S3).

**Figure 4:**
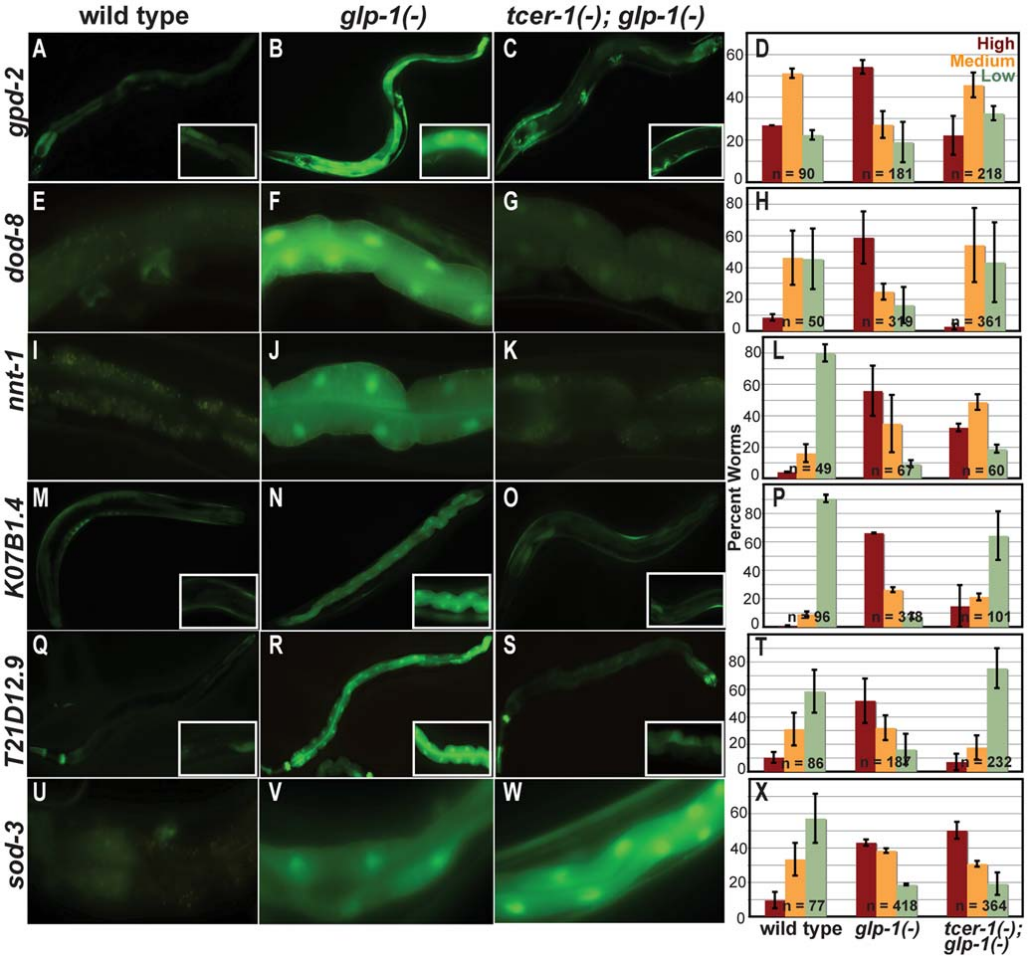
*tcer-1* is required for the up-regulation of DAF-16/FOXO-target genes in *glp-1* mutants. (A–X) Expression of DAF-16/FOXO-target genes in wild-type worms (left column), *glp-1* mutants (middle column), and *tcer-1*; *glp-1* mutants (right column). Animals were observed on day 2 of adulthood using transcriptional GFP reporters for individual genes. Bar graph representation of the data for each gene is shown in the far-right column. In each experiment, worms were classified into those with High (red bars), Medium (orange bars), and Low (green bars) GFP levels. ‘n’ signifies the total number of worms examined in two or three trials. Error bars represent the standard error of the mean. The elevated expression observed in *glp-1* mutants was reduced in the case of *gpd-2* (A–D), *dod-8* (E–H), *nnt-1* (I–L), *K07B1.4* (M–P) and *T21D12.9* (Q–T). Expression of *sod-3* was elevated further (U–X). In (A–C), (M–O), and (Q–S), images were observed at 200× magnification. The insets in these panels show intestinal cells of the same strains imaged at 400×. In (E–G), (I–K), and (U–W), images of intestinal cells were observed at 400×. For more information about the expression patterns of these genes, and other DAF-16/FOXO-targets, see Table S3.

We then asked whether the up-regulation of these new genes, as well as the known *daf-16-*dependent germline-regulated genes, required *tcer-1*. We found that this was the case for six of the nine genes we examined. The up-regulation of *gpd-2*, *dod-8*, *nnt-1*, *K07B1.4*, and *T21D12.9* was strongly attenuated in *tcer-1*; *glp-1* mutants (Figure 4A–4T) and *pssy-1* expression was moderately reduced (data not shown). In contrast, *aat-1* expression remained unaltered in two trials and showed a small increase in expression in one (data not shown). These findings suggest that loss of TCER-1 may eliminate at least a portion of DAF-16/FOXO's transcriptional response to germline depletion. Unexpectedly, *sod-3* and *F52H3.5* levels were further increased upon *tcer-1* inactivation (Figure 4U–4X and Figure S4, respectively; see Discussion).

### *tcer-1* overexpression up-regulates DAF-16/FOXO-target genes

**Figure 5:**
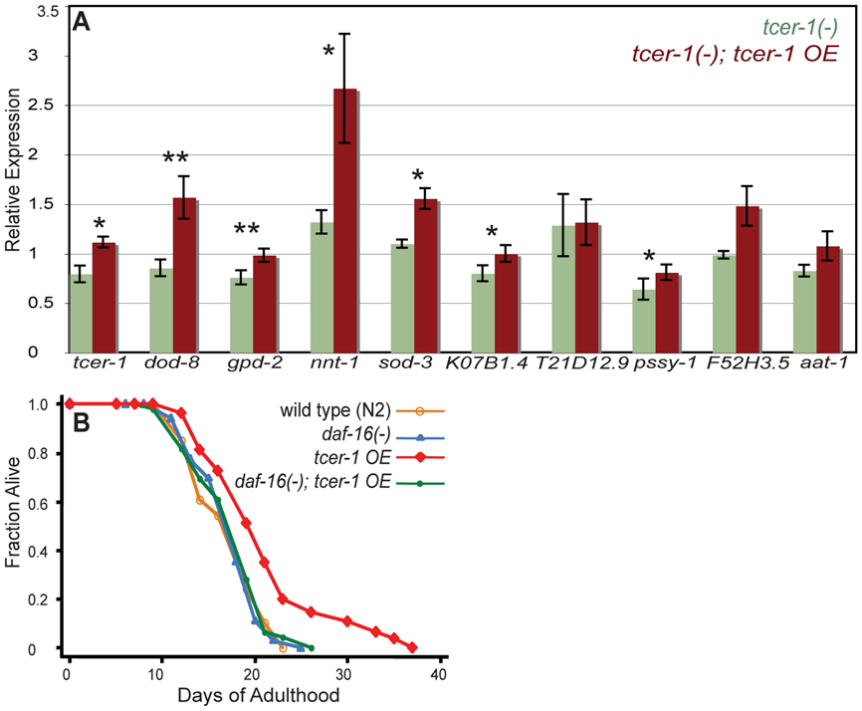
*tcer-1* overexpression causes increased transcription of DAF-16/FOXO-target genes. (A) mRNA levels of DAF-16/FOXO-targets compared between *tcer-1(−)* (green bars) and *tcer-1(−)*; *tcer-1 OE* (red bars) animals. Day 1 adults grown under similar conditions were analyzed by Q-PCR. Data shown here is combined from six independent biological replicates, each comprising 2–4 technical repeats. The genes tested are displayed on the X-axis and relative expression levels are on the Y-axis. Error bars display standard error of the mean. The asterisks represent a change in expression between the two strains with an unpaired, two-tailed *t*-test with a *P* value<0.05 (*) or <0.005 (**). (B) Lifespan extension mediated by *tcer-1* overexpression depends on *daf-16*. Wild type (N2): m = 18.4±0.3, n = 66/98; *daf-16(mu86)*: m = 17.5±0.2, n = 65/81, *P* vs. N2 = 0.03; *tcer-1 OE*: m = 21.1±0.2, n = 79/102, *P* vs. N2<0.0001; *daf-16(mu86)*; *tcer-1 OE*: m = 17.6±0.2, n = 89/104, *P* vs. N2 = 0.54, *P* vs. *daf-16(mu86)* = 0.15, *P vs. tcer-1 OE*<0.0001. See Table S4 for multiple repeats of this experiment.

As *tcer-1* reduction of function reduced the expression of many DAF-16/FOXO-target genes, we asked if TCER-1 overexpression would increase the expression of the same DAF-16/FOXO-target genes that were downregulated in *tcer-1(−)*; *glp-1(−)* mutants. Using quantitative RT-PCR (Q-PCR), we found that several such genes (*dod-8*, *gpd-2*, *nnt-1*, *K07B1.4* and *pssy-1*) showed a modest but statistically significant increase in expression when *tcer-1* was overexpressed (Figure 5A). In addition, the lifespan extension obtained by *tcer-1* overexpression required the presence of wild-type *daf-16* (Figure 5B; Table S4). Together these findings suggest that *tcer-1* overexpression may extend the lifespan of wild-type, fertile animals by stimulating expression of DAF16/FOXO-target genes that are induced in response to germline loss.

### *tcer-1* is not required for DAF-16/FOXO to extend lifespan in insulin/IGF-1 pathway mutants

**Figure 6:**
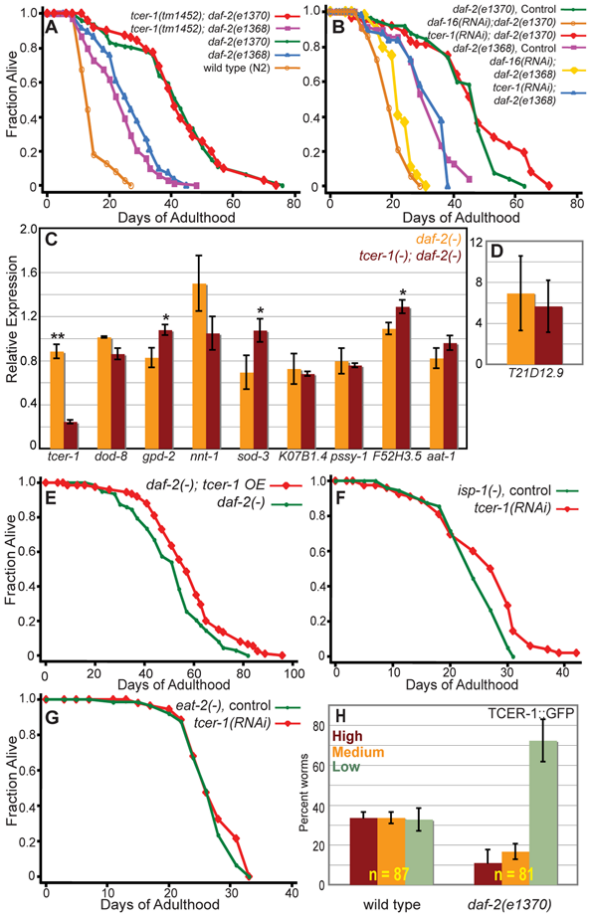
*tcer-1* reduction of function does not suppress the extended lifespans of other longevity mutants. (A) Effect of *tcer-1(tm1452)* on longevity of *daf-2(e1370)* and *daf-2(e1368)* mutants. Lifespan of *daf-2(e1370)*: m = 47.2±0.7, n = 91/103; *tcer-1(tm1452)*; *daf-2(e1370)*: m = 46.6±0.6, n = 58/102, *P* = 0.43. *daf-2(e1368)*: m = 33.1±0.4, n = 75/103; *tcer-1(tm1452)*; *daf-2(e1368)*: m = 31.3±1.8, n = 57/100, *P* = 0.1. N2: m = 16.7±0.1, n = 70/113. (B) Effect of *tcer-1* RNAi on longevity of *daf-2(e1370)* and *daf-2(e1368)* mutants. Lifespan of *daf-2(e1370)* mutants grown on bacteria containing empty control vector (m = 45.6±1.5, n = 58/77), *daf-16* RNAi bacteria (m = 16.2±0.1, n = 45/73, *P* vs. control<0.0001), and *tcer-1* RNAi bacteria (m = 47.5±2.0, n = 45/63, *P* vs. control = 0.12). *daf-2(e1368)* mutants grown on bacteria containing empty control vector (m = 31.0±1.3, n = 47/76), *daf-16* RNAi bacteria (m = 19.7±0.4, n = 35/69, *P* vs. control<0.0001) and *tcer-1* RNAi bacteria (m = 32.0±0.8, n = 41/75, *P* vs. control = 0.54). (C,D) *tcer-1(tm1452)* does not suppress the up-regulation of DAF-16/FOXO-target-genes in *daf-2(e1370)* mutants. mRNA levels of DAF-16/FOXO-regulated genes were compared between *daf-2(e1370)* mutants (orange bars) and *tcer-1(tm1452)*; *daf-2(e1370)* mutants (red bars). Day 1 adults grown under similar conditions were tested by Q-PCR analysis. The data shown here are combined from three independent biological replicates, each comprising 2–4 technical repeats. Genes tested are displayed on the X-axis and relative expression levels are on the Y-axis. Error bars display standard error of the mean. The asterisks represent a change in expression between the two strains that have a *P* value<0.01 (*) or <0.005 (**) using an unpaired, two-tailed *t*-test. The expression of *T21D12.9* has been depicted in a separate panel (D) in the interest of clarity, as this gene is expressed at higher levels than the others. (E) Effect of *tcer-1* overexpression on the lifespan of *daf-2(e1370)* mutants. *daf-2(−)*: m = 51.2±1.5, n = 63/87; *daf-2(−)*; *tcer-1 OE*: m = 57.6±1.4, n = 74/90, *P* = 0.01. See Table S2 for additional trials of this experiment. (F) Effect of *tcer-1* RNAi on longevity of *isp-1(qm150)* mutants. Lifespan of *isp-1(qm150)* mutants grown on bacteria containing empty control vector (m = 24.1±0.5, n = 39/91) and *tcer-1* RNAi bacteria (m = 26.1±0.5, n = 52/90, *P* vs. control = 0.0005). See Table S5 for multiple repeats of the lifespan experiments shown above. (G) Effect of *tcer-1* RNAi on longevity of *eat-2(ad1116)* mutants. Lifespan of *eat-2(ad1116)* mutants grown on bacteria containing empty control vector (m = 26.5±0.4, n = 59/72) and *tcer-1* RNAi bacteria (m = 27.2±0.5, n = 40/71, *P* vs. control 0.2). (H) Expression of *tcer-1* in *daf-2(e1370)* mutants. Bar graph showing reduced TCER-1::GFP expression in long-lived *daf-2(e1370)* mutants. Day 1 adults of each strain were classified into those with High (red bars), Medium (orange bars), and Low (green bars) GFP levels (focusing primarily on the intestine). ‘n’ signifies the total number of animals examined in two trials. Error bars represent the standard error of the mean.

Since *daf-16* is also required for the doubling of lifespan produced by reducing insulin/IGF-1 signaling [16],[39], we examined the role of *tcer-1* in this longevity pathway too. We found that neither *tcer-1(tm1452)* nor *tcer-1* RNAi suppressed the extended lifespans of the insulin/IGF-1-receptor mutants *daf-2(e1370)* or *daf-2(e1368)* (Figure 6A and 6B; Table S5). Therefore, TCER-1 is not part of the insulin/IGF-1 pathway. Consistent with this, we found that TCER-1 was not required in *daf-2(e1370)* mutants for the expression of any of the DAF-16/FOXO targets that were *tcer-1*-dependent in the reproductive pathway. The *tcer-1(tm1452)* mutation either had no effect, or in some cases produced a small increase in expression (Figure 6C and 6D).

Removing the germ-cell precursors of *daf-2* mutants further doubles their already long lifespan [1],[4]. Similarly, we found that overexpressing *tcer-1* in *daf-2(e1370)* mutants extended their long lifespan by another 6–12% (Figure 6E; Table S2C). In contrast, we did not observe a further extension of lifespan when *tcer-1* was overexpressed in germline-depleted worms (Figure 3F, Figure S2; Table S2C). These observations support the model that *tcer-1* functions in the germline longevity pathway but not the insulin/IGF-1 longevity pathway.

Not all longevity pathways in *C. elegans* are *daf-16* dependent. For example, the longevity response to caloric restriction caused by the feeding-defective mutant *eat-2(ad1116)* [40] is *daf-16* independent, as is the longevity response to inhibition of mitochondrial respiration caused by *isp-1(qm150)* [41]–[43]. We found that *tcer-1* was not required for either of these mutations to extend lifespan (Figure 6F and 6G; Table S5). These findings further reinforced the interpretation that TCER-1 is not a general longevity factor but instead extends lifespan specifically in response to reproductive signals.

The ability of TCER-1 overexpression to activate DAF-16/FOXO-dependent gene expression and extend lifespan suggested that the up-regulation of TCER-1 is a key switch-point by which loss of the germ cells triggers a longevity response. This up-regulation could also be part of the mechanism by which TCER-1 activity is specifically directed towards this pathway and not towards the insulin/IGF-1 pathway. To test this possibility, we examined TCER-1::GFP levels in *daf-2(e1370)* mutants. We found that *daf-2* mutants did not have elevated TCER-1::GFP levels. In contrast, TCER-1 levels were significantly reduced (Figure 6H, compare with Figure 3C). TCER-1 levels were also reduced in long-lived *eat-2(ad1116)* mutants (Figure S5), and we found that *tcer-1* overexpression further extended the lifespan of *eat-2(ad1116)* mutants (Figure S5; Table S2C). These data suggested that the specificity of TCER-1 for the reproductive pathway might be achieved, at least in part, by the regulation of its level in the animal.

### *kri-1* controls the intestinal expression of *tcer-1*

**Figure 7:**
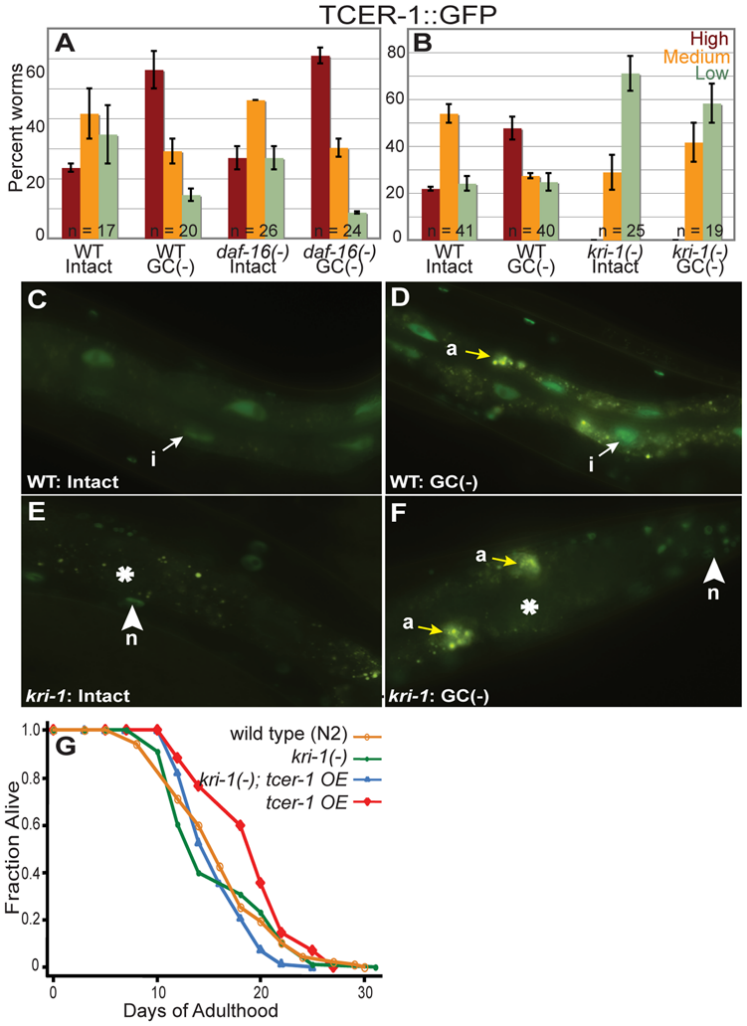
TCER-1 expression is elevated upon germline ablation in a *daf-16-*independent and *kri-1*-dependent fashion. (A) TCER-1 up-regulation does not require *daf-16*. Bar graph showing increased TCER-1::GFP levels in animals with germline-precursor cells (Z2, Z3)-ablated in a wild-type background (WT) or in *daf-16(mu86)* mutants. Day 1 adults of each strain were classified into those with High (red bars), Medium (orange bars), and Low (green bars) GFP levels (focusing primarily on the intestine). ‘n’ signifies the total number of animals examined in two trials. Error bars represent the standard error of the mean. (B–F) Intestinal TCER-1 levels are influenced by *kri-1*. TCER-1::GFP expression observed in day 1 adults of wild-type control (WT) worms (C,D) with intact gonads (C), and following germ-cell precursor (Z2, Z3)-ablation (D). Increased expression in intestinal nuclei (i) is visible (compare white arrows between (C) and (D)). Images of age-matched intact (E) or germline-ablated (F) *kri-1(ok1251)* mutants are shown. In both cases, there is drastic reduction in intestinal GFP (asterisks). In some trials, we observed a small increase in the cytoplasmic GFP levels in intestinal cells following germline ablation, but this was not quantifiable due to the strong reduction in overall GFP signal in the intestine. Autofluorescence (a) in intestinal cells is indicated by yellow arrows. *kri-1(ok1251)* did not alter TCER-1::GFP in any other tissues such as neurons (n, arrowheads). Bar graph of data shown in (C–F) is depicted in (B) (key and experimental set up same as in (A)). (G) Lifespan extension produced by *tcer-1* overexpression requires *kri-1*. N2: m = 16.5±0.3, n = 90/138; *kri-1(ok1251)*: m = 15.9±0.2, n = 69/135, *P* vs. N2 = 0.19; *tcer-1 OE*: m = 20.5±0.4, n = 97/130, *P* vs. N2 = <0.0001, *P* vs. *kri-1(ok1251)*<0.0001; *kri-1(ok1251)*; *tcer-1 OE*: m = 16.0±0.5, n = 79/128, *P* vs. N2 = 0.54, *P* vs. *kri-1(ok1251)* = 0.46, *P vs. tcer-1 OE*<0.0001. See Table S4 for multiple repeats of this experiment.

How is TCER-1 up-regulation controlled by the reproductive system? To begin to address this question, we asked whether *daf-16* itself, or genes that are known to influence DAF-16/FOXO nuclear localization in response to germline loss [13], were involved. We found that the *daf-16(mu86)* null mutation did not alter TCER-1::GFP levels in wild-type or in germline-deficient animals (Figure 7A). Likewise, neither *daf-9* nor *daf-12* mutations, both of which partially inhibit DAF-16/FOXO nuclear localization in germline-defective animals, affected TCER-1 up-regulation (data not shown). However, in *kri-1(ok1251)* mutants, nuclear TCER-1::GFP levels were severely diminished in the intestine and were not increased following germ-cell ablation (Figure 7B–7F). *kri-1(ok1251)* did not alter TCER-1::GFP levels in any other tissues (Figure 7E and 7F), consistent with KRI-1's presence only in the gastrointestinal tract [13]. Thus, DAF-16/FOXO nuclear localization and TCER-1 up-regulation share a requirement for KRI-1 in their response to germ-cell loss. As expected, *kri-1(ok1251)* also abolished the lifespan extension evoked by *tcer-1* overexpression (Figure 7G; Table S4). KRI-1 is not required for DAF-16/FOXO nuclear localization or lifespan extension in *daf-2* mutants [13]. Thus, KRI-1's involvement in TCER-1 regulation explains, at least in part, why TCER-1 function is specific for the reproductive system.

## Discussion

The finding that signals from reproductive tissues can influence the lifespan of an animal is fascinating and important because both reproduction and aging are such fundamental aspects of an animal's life history. In this study, we have shown that a predicted transcription elongation factor, TCER-1, is a key regulatory target of lifespan-extending signals from the reproductive system in *C. elegans*. TCER-1's level in the animal is sensitive to cues from the reproductive system, and when overexpressed, TCER-1 can bypass the normal requirement for germline loss and extend the lifespan of fertile animals with an intact reproductive system.

Our data indicate that TCER-1 extends lifespan by collaborating with the DAF-16/FOXO transcription factor, which is also required for the loss of germ cells to extend lifespan [1]. DAF-16/FOXO has an evolutionarily-conserved role in lifespan regulation [18],[44], and is activated in many other circumstances that increase lifespan, including reduced insulin/IGF-1 signaling [8],[11] or overexpression of the sirtuin SIR-2.1 [45], heat-shock factor HSF-1 [46], AMP kinase [47] or c-Jun N-terminal kinase [48]. How such a core, conserved longevity factor is activated by many disparate stimuli is a central question in the biology of aging. In this study, we have shown that TCER-1 plays a key role in establishing a distinct pattern of DAF-16/FOXO-dependent gene expression specifically in response to signals from the germline.

### Model for TCER-1 function

We can suggest hypotheses about the molecular function of TCER-1 from studies of its human homolog, the transcription elongation factor TCERG1 (also known as CA150) [35],[36]. TCERG1 is known to associate with elongation-competent RNA Polymerase II (RNAPII) complexes and alter the elongation efficiency of nascent mRNA transcripts [36],[49],[50]. It has also been implicated in splicing [51]–[53]. TCERG1 (like TCER-1) has a modular structure, with FF repeats that associate with the C Terminal Domain (CTD) of RNAPII, and WW domains that can be used for association with other regulatory proteins [37],[50],[51],[53], consistent with its role in regulating gene expression.

The known function of TCERG1 suggests the model that TCER-1 influences longevity by promoting transcript elongation of DAF-16/FOXO-target genes. Specifically, we propose that following germline ablation, DAF-16/FOXO accumulates in nuclei and initiates transcription of a set of downstream targets, many of which require TCER-1 for transcript elongation. In germline-ablated *tcer-1* mutants, the transcription of such genes is stalled, so lifespan extension is prevented. This hypothesis is supported by recent discoveries of transcription elongation factors that selectively influence transcription initiated by particular transcription initiation (activation) factors [54],[55]. FOXO proteins are known to be regulated by many types of covalent modifications, including phosphorylation on multiple sites, deacetlyation, and in addition by regulated proteolysis [56],[57]. To our knowledge, this is the first suggestion of DAF-16/FOXO regulation exerted at the level of transcription elongation. However, it is important to note that our experiments, which utilized GFP fusions and RT-PCR (that report on the efficiency of transcription in general and not the elongation step in particular), do not rule out the possibility that *tcer-1* acts at a different stage of transcription, such as transcription initiation.

Unexpectedly, we noticed that the expression of some DAF-16/FOXO target genes, such as *sod-3* and *F52H3.5*, was further increased in *tcer-1(−)*; *glp-1(−)* mutants (Figure 4U–4X and Figure S4). We also found that the cytoplasmic levels of DAF-16::GFP were elevated in these animals (Figure S3). One explanation for these observations is that in germline-depleted *tcer-1* mutants, the silence of many DAF-16/FOXO targets triggers a compensatory response that elevates DAF-16/FOXO levels in the cytoplasm (and possibly to some extent in the nucleus as well). For *tcer-1-*dependent genes (such as *nnt-1*), this elevated DAF-16/FOXO cannot compensate for the absence of TCER-1, and their transcription remains stalled. However, for *tcer-1*-independent genes *(*such as *sod-3)*, elevated DAF-16/FOXO levels increase transcription.

### DAF-16/FOXO activates different patterns of gene expression in response to insulin/IGF-1-pathway inhibition and germline loss

Since DAF-16/FOXO increases lifespan in response to germline loss and reduced insulin/IGF-1 signaling, a simple model would be that it activates the same downstream lifespan-extending genes in both situations. However, we found that at least some of the genes that are up-regulated in germline-defective mutants in a *tcer-1* dependent manner are not up-regulated in *daf-2* mutants (for example *T21D12.9*; see Table S3). This finding suggests that the differential deployment of *tcer-1* activity allows reproductive cues and reduced insulin/IGF-1 levels to trigger different patterns of DAF-16/FOXO-dependent gene expression. Thus, this situation illustrates how the activities of broadly-deployed transcription factors can be tailored to precise transcriptional outputs in response to specific signals. Interestingly, some genes that are up-regulated in germline-defective mutants in a *tcer-1-*dependent way are also up-regulated in *daf-2* mutants, but independently of *tcer-1* (for example, *dod-8*). Perhaps a different transcription-elongation factor, one that can substitute for TCER-1, is activated by reduction of insulin/IGF-1 activity.

### Activation of the reproductive longevity pathway in animals that have germ cells

A major question in the field of aging is whether a mode of lifespan extension that is normally triggered by one specific condition, such as caloric restriction, can be triggered instead by intervention at a downstream regulatory step of the pathway. As described above, we found that the longevity pathway activated by loss of the germ cells could potentially operate independently of signals related to reproduction. The finding that germline removal triggers the transcriptional up-regulation of *tcer-1* suggested that this regulatory step might function as a germline-pathway specific switch that activates downstream longevity processes. In fact this appears to be the case: when *tcer-1* is overexpressed in intact animals that have germ cells, lifespan is extended. This lifespan extension has no detrimental effects on progeny production (data not shown) and is accompanied by several hallmarks of the germline-longevity pathway, including a requirement for KRI-1 and DAF-16/FOXO, and the stimulation of a pattern of gene expression similar to that produced by germline removal. The lifespan increment obtained by overexpressing *tcer-1* was not as large as that produced by germ-cell loss. Nevertheless, these data, and other recent observations [58], suggest that at least some of the beneficial longevity effects produced by loss of the germline can be replicated without perturbing reproduction itself.

### Perspective

The last few decades have transformed our view of aging from a haphazard, unregulated phenomenon to a process influenced by conserved genetic pathways. The insulin/IGF-1 pathway and DAF-16/FOXO orthologs have been implicated in lifespan regulation in worms, flies, mice, dogs and several human populations [16], [26]–[33],[59],[60]. Studies in flies and mice indicate that pathways linking reproduction and aging maybe widespread in nature too. Given this context, the identification of a regulator, TCER-1, that selectively responds to reproductive signals to influence the transcriptional response of a conserved longevity-determinant, DAF-16/FOXO, is exciting. It will be interesting to learn whether TCER-1's function in aging has also been conserved during evolution.

## Materials and Methods

### Strains

Strains were maintained as described earlier [61]. The *tcer-1(tm1452)* mutant was provided by the National Bioresource Project (Japan) and outcrossed three times to the Kenyon Lab N2 stock. Transgenic strains expressing GFP under control of promoters of DAF-16/FOXO-target genes were obtained from the CGC, or from Prof. David Baillie's laboratory (*Caenorhabditis* Gene Expression Consortium). The details of these transgenic animals, strains generated using them and other strains used in this study are described in Table S6 and Table S3.

### Molecular biology

The *Ptcer-1::tcer-1::gfp* fusion construct was generated as described previously [62]. The complete coding sequence of *tcer-1* (4.1 kb) and 1.6 kb 5′ upstream promoter sequence were amplified using the following primers: Forward- 5′ GCA AGT ATT TGA GCA CTA CTG TCA AGG GC 3′, Reverse- 5′ *AGT CGA CCT GCA GGC ATG CAA GCT* TTG CTT TCT GCG ATC CCG CTC 3′. *The gfp construct (1.9 kb) was amplified from the vector pPD95.75 using the following primers: Forward- 5′ AGC TTG CAT GCC TGC AGG TCG ACT 3′, Reverse- 5′ AAG GGC CCG TAC GGC CGA CTA GTA GG 3′. The complete gfp fusion construct (7.6 kb) was amplified by pooling the products of the above two PCRs and using the following primers: Forward: 5′ GCC GGT CAT GCT CTT CTT CAA C 3′, Reverse- 5′ GGA AAC AGT TAT GTT TGG TAT ATT GGG AAT GTA TTC TG 3′. The co-injection marker Podr-1::rfp was amplified from the plasmid pCF155.*

### Transgenic strains

To generate the TCER-1::GFP expressing worms, *Ptcer-1::tcer-1::gfp* was injected as described earlier [63] at 50 ng/µl or 20 ng/µl, along with the co-injection marker *Podr-1::rfp* (75 ng/µl), into N2 worms (see Table S6). As a control, *Podr-1::rfp* alone was injected into N2 worms (75 ng/µl). The resulting transgenic control strains (CF2144, CF2145) had mean lifespans that were the same as N2.

### Lifespan analysis

Lifespan assays in general were conducted as previously described [64]. For *glp-1* mutant lifespan assays, eggs were incubated at 20°C for 2–6 hrs, transferred to 25°C to eliminate germ cells, then shifted back to 20°C on day 1 of adulthood for the rest of their lifespan. All other lifespan assays were performed at 20°C. In all cases, the L4 stage was counted as day 0 of adulthood. In all experiments with TCER-1::GFP, worms were examined for *Podr-1::rfp* co-injection marker under a Leica MZ16F stereomicroscope (Wetzlar, Germany) on day 0 and isolated for lifespan experiments. The non-transgene carrying siblings were used as negative controls in the same experiment. *glp-1* mutant strains used in lifespan assays were completely sterile. Fertile strains were transferred every other day to fresh plates until progeny production ceased. Animals that crawled off the plate, exploded, bagged, or became contaminated were censored. Stata 10.0 and 8.2 (Stata Corporation, Texas, USA) and (for some lifespans) Statview 5.0.1 (SAS) softwares were used to calculate mean life spans and perform statistical analyses. *P* values were determined using log-rank (Mantel-Cox) statistics.

### RNAi experiments

We screened a *C. elegans* chromosome II RNAi library to identify RNAi clones that suppressed the extended lifespan of *glp-1* mutants. Details of a similar screen were described earlier [13]. All RNAi experiments were performed as described previously [65],[66]. In general, RNAi clones were inoculated overnight at 37°C in LB medium containing 10 µg/ml tetracycline and 100 µg/ml carbenicillin, and seeded onto NG-carbenicilin plates supplemented with 0.1 M IPTG. For all lifespan experiments, and the RNAi screen, worms were exposed to RNAi clones from hatching. All RNAi clones were confirmed by sequencing (M13-forward primer) and upon start of every experiment, by PCR (T7 primers). Clones were obtained from the libraries described previously [65],[66]. pAD12 (empty vector) was used as the negative control, and pAD43 (*daf-16* RNAi) as the positive control for the screen and for individual lifespans [43]. The *tcer-1* RNAi clone also targets a partial duplication of *tcer-1*, the gene *ZK1127.6*, whose predicted protein product contains only FF domains. Transcriptional and full-length translational GFP-tagged reporters for *ZK1127.6* showed no basal expression or induction on germline ablation suggesting that it is a non-functional duplication of *tcer-1* (*ZK1127.9*).

### Laser ablations

Laser ablations of germ-cell precursors (Z2, Z3) were performed using a Zeiss Axiophot with a laser attachment (Photonics Instruments, USA) as described earlier [67]. Briefly, eggs were transferred to fresh plates and left at 20°C for 1–3 hrs. Hatched L1 larvae were mounted on slides with 2% Agarose pads containing 1.5 mM Sodium Azide anesthetic. As intact controls, worms were subjected to all the steps of this process except for ablation. Ablated worms (and controls) were removed from the slide and grown at 20°C until required for GFP assays or lifespan analysis.

### GFP assays

Eggs of worms carrying the *Pdaf-16::daf-16::gfp* reporter construct and the different DAF-16/FOXO-target gene reporters (in wild-type, *glp-1* mutant and *tcer-1*; *glp-1* mutant backgrounds) were incubated at 20°C for 2–6 hrs, transferred to 25°C to eliminate germ cells, then shifted back to 20°C on day 1 of adulthood. GFP assays were conducted on day 2 of adulthood, using a Leica MZ16F (Wetzlar, Germany) stereomicroscope with standard fluorescence filter sets. All assays were performed blind after initial familiarization of GFP levels in control plates by the experimenter.

### Microscopy

All fluorescence images were captured using a Retiga EXi Fast1394 CCD digital camera (QImaging, Burnaby, BC, Canada) attached to a Zeiss Axioplan 2 compound microscope (Zeiss Corporation, Jena, Germany). Openlab 4.0.2 software (Improvision, Coventry, U.K.) was used for image acquisition. GFP assays were conducted on a Leica MZ16F (Wetzlar, Germany) stereomicroscope with fluorescence filter sets or (in the case of *Pnnt-1::gfp*) the Zeiss Axioplan 2 compound microscope mentioned above. Preliminary image processing was performed using Photoshop 10 (Adobe Creative Suite 3, USA).

### Q-PCRs

Total RNA was isolated from synchronized populations of approximately 15,000 day 1 *daf-2* and *tcer-1*; *daf-2* mutants. For comparing, *tcer-1(−) and tcer-1(−)*; *tcer-1 OE* strains, worms were picked manually for RNA isolation. Eggs were transferred to fresh plates. Day 0 (L4) transgenic animals carrying *tcer-1(−)*; *tcer-1 OE* were isolated using a Leica MZ16F stereomicroscope with standard fluorescence filters. 2,000–5,000 worms were picked manually for RNA isolation for each biological replicate. A similar number of *tcer-1* mutants were also collected manually. Both strains were allowed to grow for 24 hrs and on day 1 of adulthood used for RNA isolation. Total RNA was extracted using TRIzol reagent (Invitrogen) and purified using Qiagen RNAeasy Mini Kit. cDNA was generated using Protoscript First Strand cDNA Synthesis Kit (New England Biolabs). SybrGreen real-time Q-PCR reactions were performed on an Applied Biosystems 7300 Real Time PCR System. The primers used in this study are listed in Table S7.

## Supporting Information

Figure S1 Effect of *tcer-1* RNAi on TCER-1::GFP expression.
(1.37 MB TIF)

Figure S2 Effect of *tcer-1* overexpression on the lifespan of germline-precursor (Z2, Z3) ablated worms.
(0.48 MB TIF)

Figure S3 DAF-16/FOXO nuclear localization in germline-ablated *tcer-1(tm1452)* mutants.
(0.86 MB TIF)

Figure S4 Expression of *F52H3.5* in wild type, *glp-1* mutant, and *tcer-1*; *glp-1* mutant backgrounds.
(2.08 MB TIF)

Figure S5 Role of *tcer-1* in the longevity of mutants in *daf-16*-independent pathways.
(0.39 MB TIF)

Table S1 Effect of *tcer-1* RNAi and mutation on the lifespans of *glp-1* mutants and wild-type worms.
(0.15 MB PDF)

Table S2 Effect of *tcer-1* overexpression on the lifespan of wild-type animals, *tcer-1* and long-lived mutants.
(0.14 MB PDF)

Table S3 Expression of DAF-16/FOXO-target genes in wild-type animals, *glp-1* and *daf-2* mutants.
(0.15 MB PDF)

Table S4 Effect of *daf-16(mu86)* and *kri-1(ok1251)* mutations on lifespan extension induced by *tcer-1* overexpression.
(0.11 MB PDF)

Table S5 Effect of *tcer-1* reduction of function on the lifespans of other long-lived mutants.
(0.11 MB PDF)

Table S6 Strains used in this study.
(0.12 MB PDF)

Table S7 Q-PCR primers used in this study.
(0.07 MB PDF)
