# Supplementary figures and images for: A Transcription Elongation Factor That Links Signals from the Reproductive System to Lifespan Extension in Caenorhabditis elegans
Source: PLoS Genet. 2009 Sep 11;5(9):e1000639. doi: 10.1371/journal.pgen.1000639 (PMC2729384; doi:10.1371/journal.pgen.1000639)

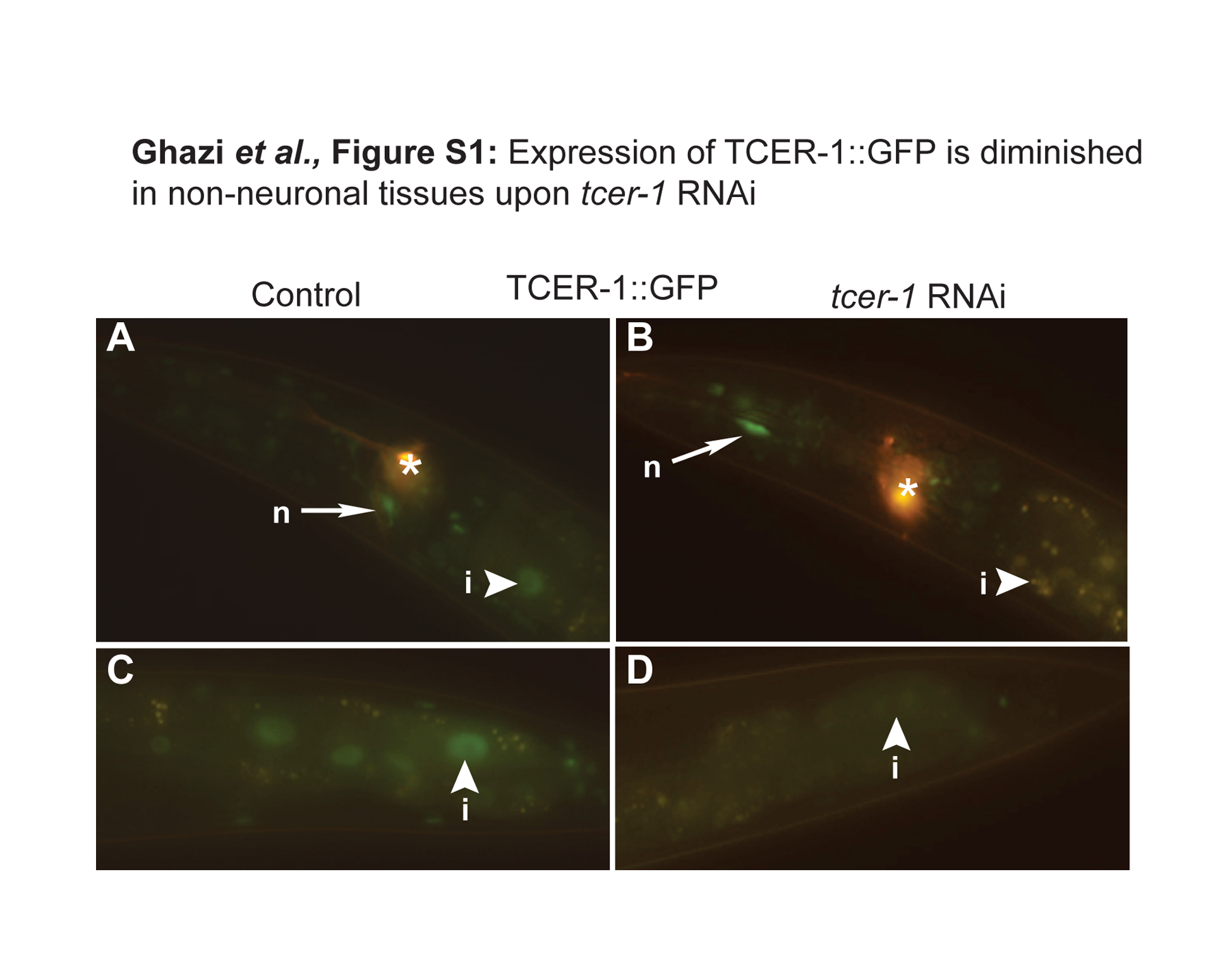

Supplement: Figure S1 — Effect of tcer-1 RNAi on TCER-1::GFP expression. (1.37 MB TIF) [file pgen.1000639.s001.tif]

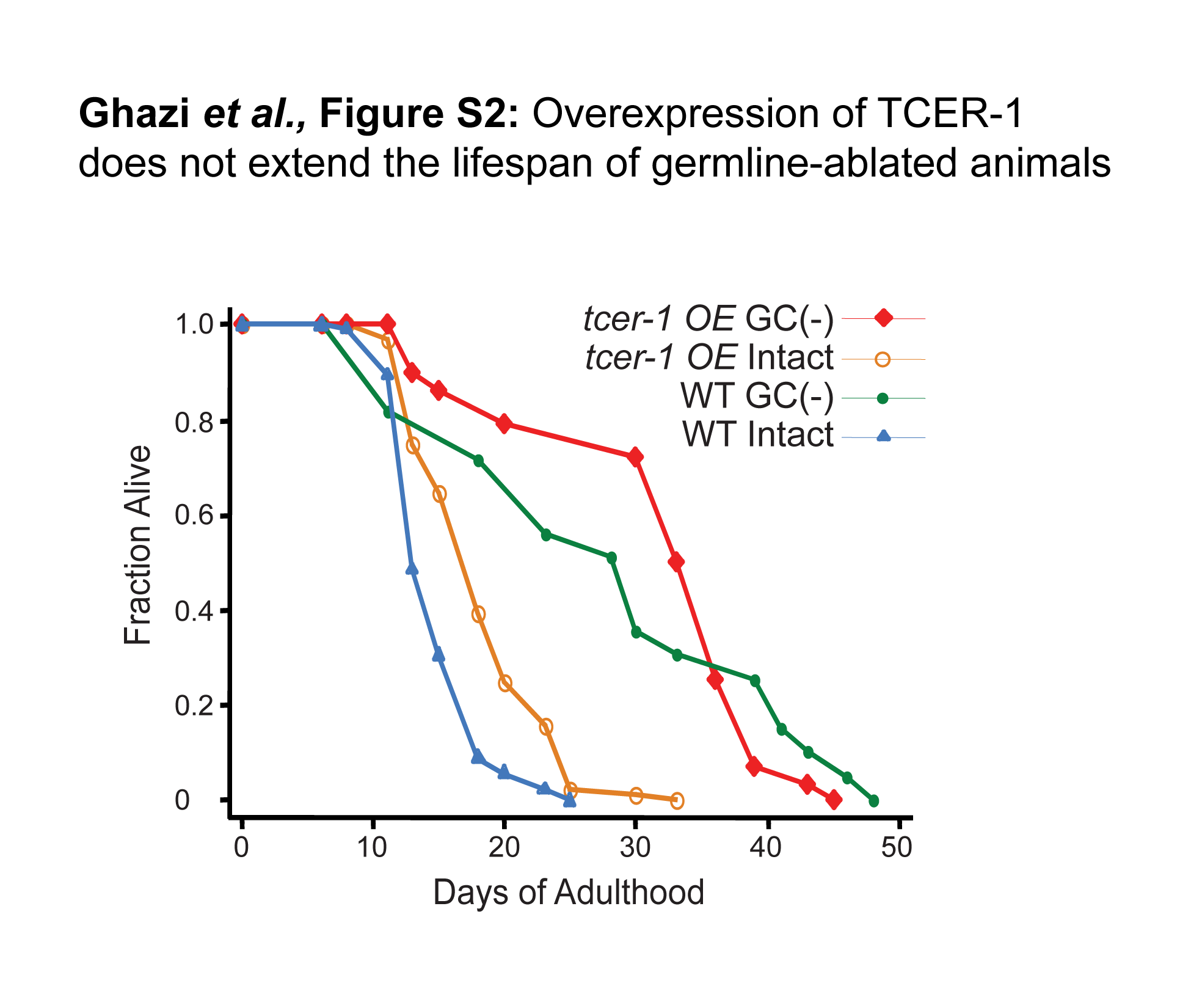

Supplement: Figure S2 — Effect of tcer-1 overexpression on the lifespan of germline-precursor (Z2, Z3) ablated worms. (0.48 MB TIF) [file pgen.1000639.s002.tif]

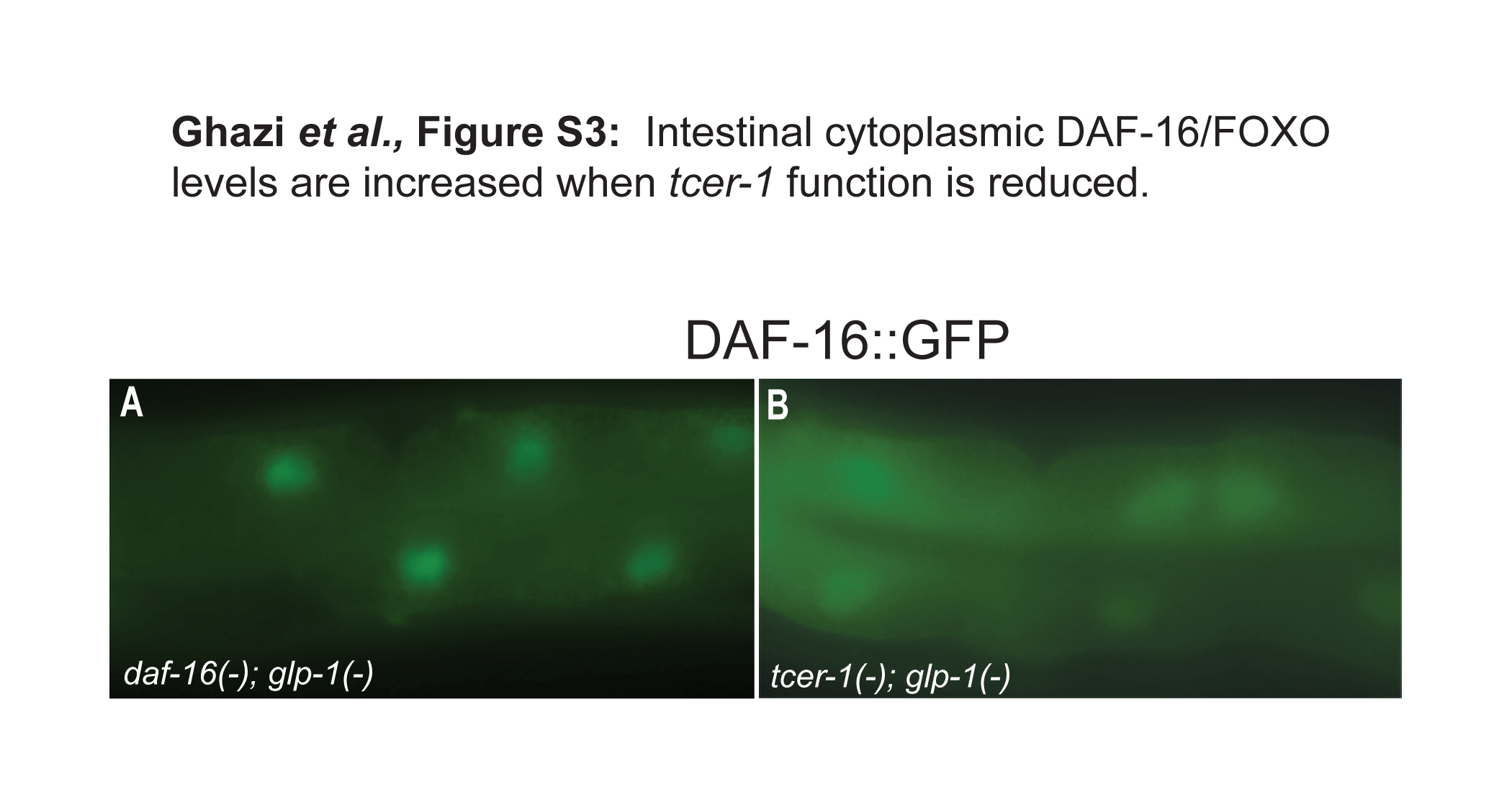

Supplement: Figure S3 — DAF-16/FOXO nuclear localization in germline-ablated tcer-1(tm1452) mutants. (0.86 MB TIF) [file pgen.1000639.s003.tif]

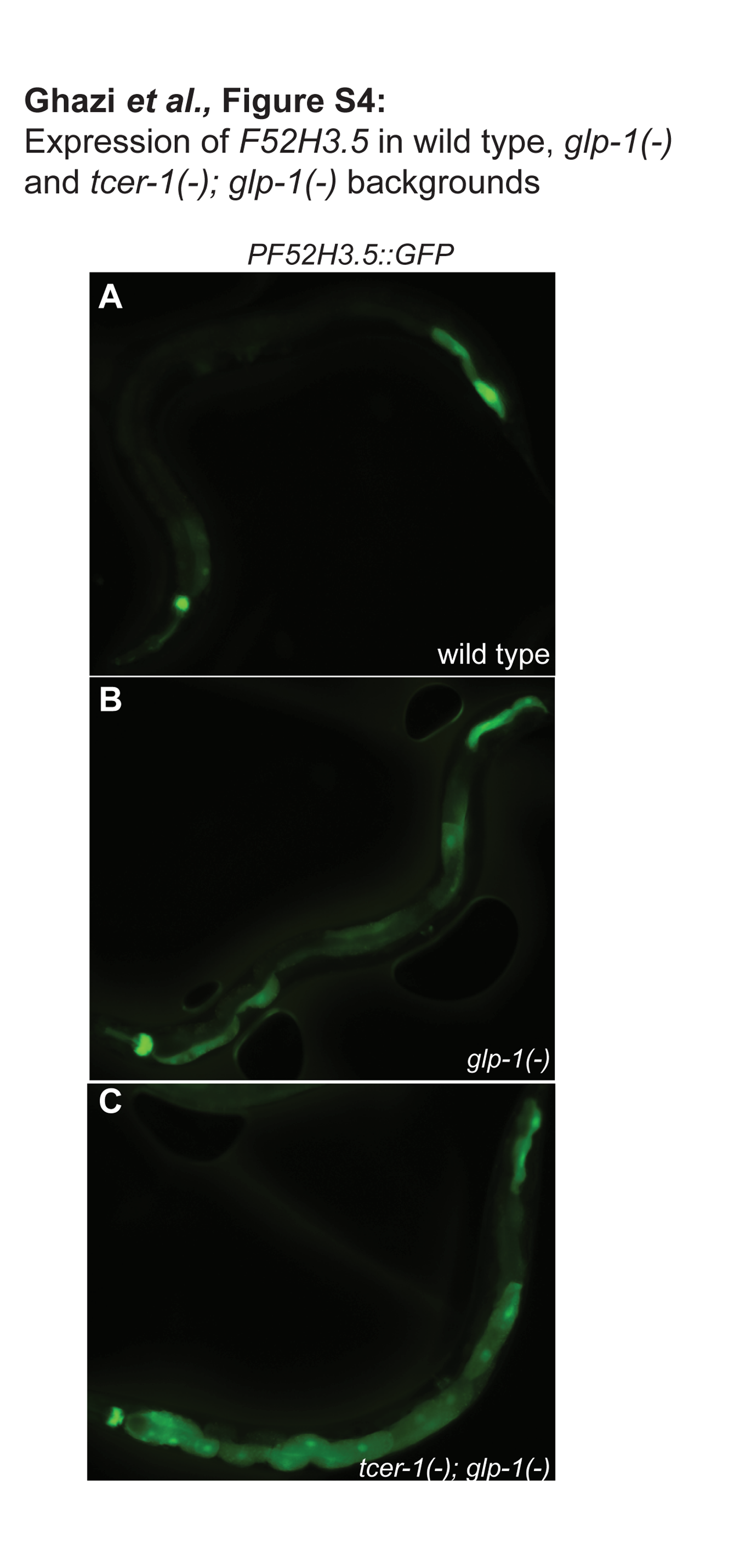

Supplement: Figure S4 — Expression of F52H3.5 in wild type, glp-1 mutant, and tcer-1; glp-1 mutant backgrounds. (2.08 MB TIF) [file pgen.1000639.s004.tif]

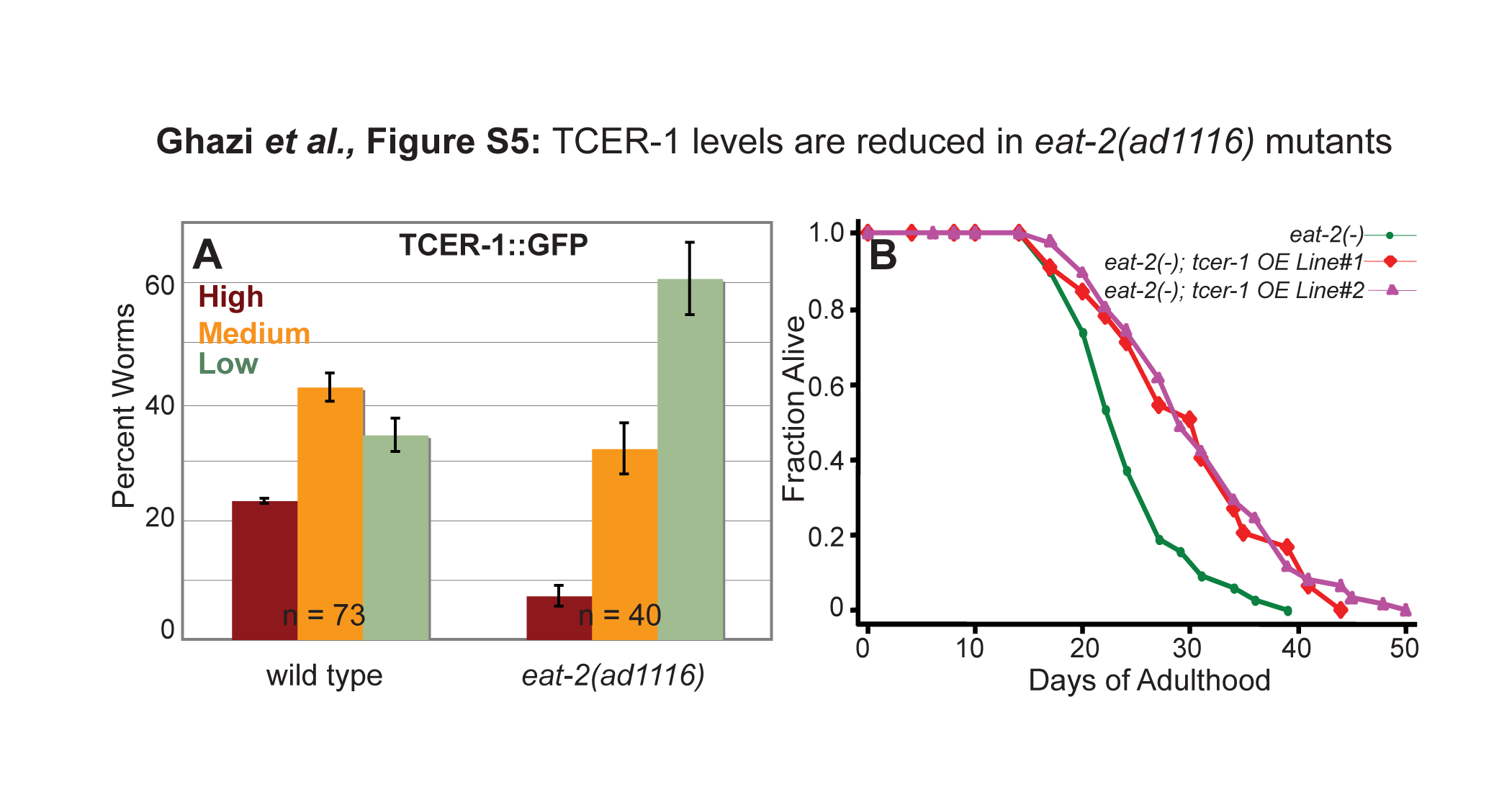

Supplement: Figure S5 — Role of tcer-1 in the longevity of mutants in daf-16-independent pathways. (0.39 MB TIF) [file pgen.1000639.s005.tif]
