# Supplementary material for: A Transcription Elongation Factor That Links Signals from the Reproductive System to Lifespan Extension in Caenorhabditis elegans
Source: PLoS Genet. 2009 Sep 11;5(9):e1000639. doi: 10.1371/journal.pgen.1000639 (PMC2729384; doi:10.1371/journal.pgen.1000639)
Supplement: Table S1 — Effect of tcer-1 RNAi and mutation on the lifespans of glp-1 mutants and wild-type worms. (0.15 MB PDF) [file pgen.1000639.s006.pdf]

Table S1: Effects of *tcer-1* reduction of function on the lifespan of wild-type worms and long-lived *glp-1* mutants.

**Table S1. A: Effects of *tcer-1* RNAi on lifespan**							
**Genotype**	**RNAi treatment**	**Trial**	**Mean LS ± SEM (days)**	**Events/ Obs** [a]	**% of control lifespan**	**P *vs.* RNAi control** [b]	**P *vs.* *daf-16* RNAi**
glp-1(-)	Control	1	23.7 ± 0.7	84/89			
	tcer-1		16.3 ± 0.4	80/90	-31.2	<0.0001	
	daf-16		15.3 ± 0.3	88/92	-35.4	<0.0001	
	Control	2*, $	27.0 ± 1.1	112/114			
	tcer-1		18.5 ± 0.3	119/122	-31.4	<0.0001	<0.0001
	tcer-1(MV)		18.1 ± 0.4	96/114	-32.2	<0.0001	<0.0001
	daf-16		15.2 ± 0.2	110/113	-43.7	<0.0001	
	Control	3	24.5 ± 0.7	112/114			
	tcer-1		19.9 ± 0.4	96/109	-18.7	<0.0001	<0.0001
	tcer-1(MV)		18.2 ± 0.4	103/124	-25.7	<0.0001	0.02
	daf-16		16.9 ± 0.4	61/67	-31.0	<0.0001	
	Control	4	20.0 ± 0.7	98/105			
	tcer-1		13.9 ± 0.2	102/105	-30.5	<0.0001	0.04
	daf-16		12.9 ± 0.3	98/105	-35.5	<0.0001	
	Control	5$$	23.6 ± 1.0	83/95			
	tcer-1		18.1 ± 0.5	101/102	-23.3	<0.0001	<0.0001
	tcer-1(MV)		17.7 ± 0.5	56/98	-25.0	<0.0001	<0.0001
	daf-16		14.2 ± 0.2	86/89	-39.8	<0.0001	
	Control	6	21.7 ± 1.0	86/90			
	tcer-1		18.2 ± 0.5	71/72	-16.1	<0.0001	<0.0001
	daf-16		15.3 ± 0.3	87/89	-29.4	<0.0001	
	Control	7	19.8 ± 1.2	78/84			
	tcer-1		15.1 ± 0.5	71/72	-23.7	<0.0001	<0.0001
	daf-16		12.9 ± 0.4	41/72	-34.8	<0.0001	
	Control	8	21.4 ± 0.9	79/83			
	tcer-1		18.2 ± 0.3	86/90	-14.9	<0.0001	<0.0001
	daf-16		15.3 ± 0.3	87/89	-28.5	<0.0001	

N2	Control	1**, [$]	19.9 ± 0.6	49/72			
	tcer-1		18.7 ± 0.5	66/72	-6.0	0.2	0.002
	daf-16		16.7 ± 0.5	62/72	-16.0	<0.0001	
	Control*	2	19.5 ± 0.3	81/99			
	tcer-1		19.0 ± 0.4	49/90	-2.6	0.25	0.0001
	daf-16		16.7 ± 0.3	76/88	-14.4	<0.0001	
	Control	4	20.8 ± 0.6	80/112			
	tcer-1		19.1 ± 0.6	87/105	-3.6	0.07	0.001
	daf-16		17.0 ± 0.4	74/112	-18.2	<0.0001	
	Control	5[$$]	19.5 ± 0.5	70/92			
	tcer-1		17.2 ± 0.4	77/90	-11.7	<0.0001	
	daf-16		16.7 ± 0.4	76/89	-14.3	<0.0001	0.63

Table S1. B: Effect of *tcer-1(-)* on lifespan of *glp-1(-)* mutants

**Genotype**	**Trial**	**Mean LS ± SEM (days)**	**Events/ Obs[a]**	**% of *glp-1(-)* control lifespan**	**P *vs. glp-1(-)***
glp-1(-)	1	31.1 ± 0.7	204/241		
CF2152<br>*tcer-1(-); glp-1(-) Line# 1.1-11.5*		25.6 ± 0.9	94/120	-17.6	<0.0001
CF2153<br>*tcer-1(-); glp-1(-) Line# 1.1-4.5*		28.0 ± 0.9	105/120	-9.9	0.001
CF2154<br>*tcer-1(-); glp-1(-) Line# 1.1-11.1*		23.7 ± 0.9	81/119	-23.7	<0.0001
CF2155<br>*tcer-1(-); glp-1(-) Line# 14.1-12.5*		27.0 ± 0.9	96/120	-13.1	<0.0001
*glp-1(-)****	2[$$$]	32.9 ± 0.3	233/258		
CF2152		23.9 ± 0.9	90/120	-28.0	<0.0001
CF2153		31.1 ± 0.9	116/134	-6.3	0.06
CF2154***		21.8 ± 0.5	77/115	-34.3	<0.0001
CF2155		25.7 ± 0.8	116/135	-22.5	<0.0001
glp-1(-)	3	29.5 ± 0.4	166/195		
CF2152		22.5 ± 1.2	76/91	-23.7	<0.0001

CF2153		32.3 ± 1.5	98/121	±9.4	0.004
CF2154		25.0 ± 1.3	75/116	-15.2	0.0007
CF2155		24.5 ± 1.0	86/107	-16.9	<0.0001
glp-1(-)	4	30.1 ± 1.1	102/103		
CF2152		23.5 ± 1.1	81/102	-21.9	0.0003
CF2153		27.1 ± 0.8	103/107	-9.9	0.009
CF2154		24.3 ± 1.0	89/99	-19.2	0.0003
CF2155		21.1 ± 0.8	82/90	-29.9	<0.0001

Table S1. C: Rescue of *tcer-1(-); glp-1(-)* shortened lifespan by TCER-1::GFP *(tcer-1 OE)*

Genotype	Trial	Mean LS ± SEM (days)	Events/ Obs[a]	P *vs* Control	P *vs* CF2154
N2	1	18.6 ± 0.4	61/96		
CF2166[#]<br>*tcer-1(-) Line#8*		19.1 ± 0.2	50/64	0.28<br>(*vs* N2)	
CF2167<br>*tcer-1(-) Line#9*		19.8 ± 0.6	68/81	0.08<br>(*vs* N2)	
glp-1(-)[#]		31.1 ± 0.7	204/241		
CF2154[#]<br>*tcer-1(-); glp-1(-)*		23.4 ± 0.5	81/119	<0.0001<br>(*vs glp-1*)	
CF2859a[#]<br>*tcer-1(-); glp-1(-); tcer-1 OE Line #24*		32.4 ± 0.3	111/116	0.5<br>(*vs glp-1*)	<0.0001
CF2859b<br>*tcer-1(-); glp-1(-); tcer-1 OE Line #81*		30.9 ± 0.8	96/123	0.42<br>(*vs glp-1*)	<0.0001
N2	2	18.5 ± 0.5	94/116		
CF2166		18.6 ± 0.5	91/108	0.63<br>(*vs* N2)	
CF2167		19.4 ± 0.6	91/105	0.1<br>(*vs* N2)	
glp-1(-)		33.2 ± 0.7	233/258		
CF2154		21.8 ± 1.0	77/115	<0.0001<br>(*vs glp-1*)	
CF2859a		27.9 ± 0.8	100/102	<0.0001<br>(*vs glp-1*)	0.0003

CF2859b		25.8 ± 1.2	67/72	<0.0001 *(vs glp-1)*	0.02
CF2859c *tcer-1(-); glp-1(-); tcer-1 OE Line #91*		28.8 ± 1.1	85/89	0.003 (*vs glp-1*)	<0.0001
N2***	1[$$$]	18.9 ± 0.5	60/121		
CF2166***		19.0 ± 0.4	96/110	0.84	
CF2167		18.3 ± 0.4	48/120	0.93	
N2	2	17.0 ± 0.3	75/89		
CF2166		13.9 ± 0.4	65/89	<0.0001	
CF2167		13.6 ± 0.6	70/83	<0.0001	

[a] Some animals were censored as described in Materials and Methods.

[b] RNAi control refers to worms exposed to empty vector plasmid without an RNAi insert.

LS: lifespan, SEM: standard error of the mean.

MV refers to clones obtained from the RNAi library described earlier [1]. All other clones were obtained from the Ahringer collection [2].

*, **, *** Experiments depicted in Figures 1A, 1B and 1C, respectively.

[#] Experiment depicted in Figure 2D.

[$, $$, $$$] Experiments performed simultaneously.

References:

1. Lamesch, P, Milstein, S, Hao, T, Rosenberg, J, Li N, *et al.* (2004) *C. elegans* ORFeome version 3.1: increasing the coverage of ORFeome resources with improved gene predictions. Genome Res 14: 2064-2069.

2. Kamath, RS, Fraser, AG, Dong, Y, Poulin, G, Durbin, R *et al.* (2003) Systematic functional analysis of the *Caenorhabditis elegans* genome using RNAi. Nature 421: 231-237.
