# Supplementary material for: A Transcription Elongation Factor That Links Signals from the Reproductive System to Lifespan Extension in Caenorhabditis elegans
Source: PLoS Genet. 2009 Sep 11;5(9):e1000639. doi: 10.1371/journal.pgen.1000639 (PMC2729384; doi:10.1371/journal.pgen.1000639)
Supplement: Table S2 — Effect of tcer-1 overexpression on the lifespan of wild-type animals, tcer-1 and long-lived mutants. (0.14 MB PDF) [file pgen.1000639.s007.pdf]

<u>Table S2:</u> Effect of *tcer-1* overexpression on the lifespan of wild-type worms and long-lived mutants.

**Table S2. A: Effect of *tcer-1* overexpression on lifespan of wild-type worms**					
**Genotype**	**Trial**	**Mean LS ± SEM (days)**	**Events/ Obs[a]**	**% Effect on control**	**P vs. control**
CF2144*,[@] *(Podr-1::RFP) muEx321* Co-injection marker (wild type) control	1	17.4 ± 0.4	81/89		
CF2032* *(Ptcer-1::tcer-1::GFP, Podr-1::RFP) muEx307*		21.2 ± 0.4	85/90	+21.8	<0.0001
CF2033 *(Ptcer-1::tcer-1::GFP, Podr-1::RFP) muEx308*		19.9 ± 0.5	89/100	+14.3	0.001
CF2148 *(Ptcer-1::tcer-1::GFP, Podr-1::RFP) muEx325*		20.5 ± 0.5	71/78	+17.8	<0.0001
CF2149* *(Ptcer-1::tcer-1::GFP, Podr-1::RFP) muEx326*		21.6 ± 0.6	74/79	+24.1	0.0002
CF2150 *(Ptcer-1::tcer-1::GFP, Podr-1::RFP) muEx327*		20.9 ± 0.5	69/87	+20.1	<0.0001
CF2151 *(Ptcer-1::tcer-1::GFP, Podr-1::RFP) muEx328*		20.3 ± 0.5	82/92	+16.6	<0.0001
CF2144 (Wild-type Control)	2	16.5 ± 0.5	82/87		
CF2148		20.5 ± 0.7	64/67	+24.2	<0.0001
CF2149		18.3 ± 0.8	71/83	+10.9	0.03
CF2150		18.1 ± 0.6	60/66	+9.6	0.05
CF2151		17.7 ± 0.6	56/64	+7.2	0.17
N2 (Wild-type control)	3	16.9 ± 0.5	56/68		
CF2031 *(Ptcer-1::tcer-1::GFP, Podr-1::RFP) muEx306*		18.8 ± 0.6	84/95	+11.2	0.01
CF2032		19.4 ± 0.5	84/95	+14.7	0.001
CF2033		19.4 ± 0.6	99/110	+14.7	0.001
CF2034 *(Ptcer-1::tcer-1::GFP, Podr-1::RFP) muEx309*		17.5 ± 0.6	99/110	+3.5	0.32

**Table S2. B: Effect of *tcer-1* overexpression on lifespan of *tcer-1(tm1452)* mutants**						
**Genotype**	**Trial**	**Mean LS ± SEM (days)**	**Events/ Obs[a]**	**% effect on control**	**P vs N2**	**P vs CF2032**
N2	1	18.6 ± 0.4	60/96			
CF2166<br>*tcer-1(-) Line #8*		19.1 ± 0.6	50/64	+2.6	0.28	
CF2167<br>*tcer-1(-) Line #9*		19.8 ± 0.6	68/81	+6.4	0.08	
CF2032<br>*(Ptcer-1::tcer-1::GFP, Podr-1::RFP)*		21.5 ± 0.7	76/111	+15.5	0.0001	
CF2857<br>*tcer-1(-); Ptcer-1::tcer-1::GFP, Podr-1::RFP*<br>*Line #16*		23.8 ± 0.6	96/103	+27.9	<0.0001	0.04
CF2858<br>*tcer-1(-); Ptcer-1::tcer-1::GFP, Podr-1::RFP*<br>*Line #18*		21.5 ± 0.6	82/93	+15.5	0.0004	0.9
N2	2	18.5 ± 0.5	94/116			
CF2166**<br>*tcer-1(-) Line #8*		18.6 ± 0.4	91/108	ne	0.63	
CF2167<br>*tcer-1(-) Line #9*		19.4 ± 0.6	91/105	+4.8	0.1	
CF2032<br>*(Ptcer-1::tcer-1::GFP, Podr-1::RFP)*		21.2 ± 0.5	78/112	+14.5	0.0004	
CF2857**<br>*tcer-1(-); Ptcer-1::tcer-1::GFP, Podr-1::RFP*<br>*Line #16*		24.6 ± 0.8	81/106	+32.9	<0.0001	0.0009
CF2858<br>*tcer-1(-); Ptcer-1::tcer-1::GFP, Podr-1::RFP*<br>*Line #18*		23.2 ± 0.8	95/114	+25.4	<0.0001	0.02

**Table S2. C: Effect of *tcer-1* overexpression on lifespan of long-lived mutants**				
**Genotype**	**Mean LS ± SEM (days)**	**Events/ Obs**[a]	**P vs. Z2, Z3-ablated or *glp-1* control**	**P vs. Intact gonad control**
CF2032[#] *tcer-1 OE* Intact Gonad Control	18.3 ± 0.4	98/115		
CF2032[#] *tcer-1 OE,* **Non transgenic siblings** Intact Gonad Control	15.0 ± 0.3	89/107		<0.0001 (vs CF2032 Intact Gonad)
CF2032[#] *tcer-1 OE* Z2, Z3 ablated	30.9 ± 2.0	29/33		
CF2032[#] *tcer-1 OE,* **Non transgenic siblings** Z2, Z3 ablated	28.2 ± 0.7	20/25	0.96 (vs CF2032 Z2, Z3 Ablated)	
CF2032 *tcer-1 OE* Intact Gonad Control	16.7 ± 0.5	39/46		
CF2032 *tcer-1 OE,* **Non transgenic siblings** Intact Gonad Control	15.3 ± 0.4	55/66		0.03 (vs CF2032 Intact gonad)
CF2032 *tcer-1 OE* Z2, Z3 ablated	30.6 ± 1.2	92/106		
CF2032 *tcer-1 OE,* **Non transgenic siblings** Z2, Z3 ablated	29.7 ± 1.6	107/120	0.76 (vs CF2032 Z2, Z3 ablated)	
glp-1(-) Control	31.3 ± 0.7	102/103		
CF2175 *glp-1(-); tcer-1 OE muEx307 Line # 32-10*	33.2 ± 1.1	82/91	0.002	
CF2177 *glp-1(-); tcer-1 OE muEx307 Line # 32-40*	30.7 ± 1.1	88/90	0.11	
CF2178 *glp-1(-); tcer-1 OE muEx308 Line # 33-16*	31.6 ± 1.0	87/90	0.13	
CF2179 *glp-1(-); tcer-1 OE muEx308 Line # 33-10*	31.7 ± 1.1	84/91	0.08	
CF2180 *glp-1(-); tcer-1 OE muEx308*	31.5 ± 0.9	81/84	0.36	

Line # 33-17				
glp-1(-) Control[***]	30.6 ± 0.2	102/104		
CF2178	31.8 ± 0.5	88/91	0.1	
CF2178<br>**Non-transgenic control siblings**	30.8 ± 0.4	97/98	0.3	0.89<br>(vs. CF2178)
CF2179[***]	30.4 ± 0.3	82/89	0.3	
CF2179[***]<br>**Non-transgenic control siblings**	30.6 ± 0.2	86/89	0.4	0.2<br>(vs. CF2179)
daf-2(e1370)	52.4 ± 1.5	95/116		
daf-2(e1370); tcer-1 OE Line#10	55.8 ± 1.6	77/120	0.01	
daf-2(e1370); tcer-1 OE Line#10[$]<br>**Non-transgenic control siblings**	51.2 ± 1.5	63/87		
daf-2(e1370); tcer-1 OE Line#10[$]	57.6 ± 1.4	74/90	0.01	
eat-2(ad1116)[$$]	24.5 ± 0.4	34/102		
eat-2(ad1116); tcer-1 OE[$$] *Line #1*	30.1 ± 0.9	30/60	0.001	
eat-2(ad1116); tcer-1[$$] *OE Line #2*	30.9 ± 0.9	64/129	<0.0001	

LS: Lifespan; SEM: Standard error of the mean

[a] Some animals were censored as described in Materials and Methods.

[@] The co-injection marker *Podr-1::RFP* did not have any effect on lifespan (CF2144 vs. N2 P = 0.24)

ne: no effect

*, **, ***: Experiments depicted in Figure 3D- F, respectively.

[#] : Experiments depicted in Figure S2.

[$, $$]: Experiments depicted in Figure 6E and Figure S5. B, respectively.
