# Supplementary material for: A Transcription Elongation Factor That Links Signals from the Reproductive System to Lifespan Extension in Caenorhabditis elegans
Source: PLoS Genet. 2009 Sep 11;5(9):e1000639. doi: 10.1371/journal.pgen.1000639 (PMC2729384; doi:10.1371/journal.pgen.1000639)
Supplement: Table S3 — Expression of DAF-16/FOXO-target genes in wild-type animals, glp-1 and daf-2 mutants. (0.15 MB PDF) [file pgen.1000639.s008.pdf]

**Table S3: Expression of DAF-16/FOXO-target genes in wild-type background, germline-ablated worms and *daf-2(e1370)* mutants.**

| GENE | COSMID | STRAIN | Wild Type Expression | Expression upon Z2, Z3 ablation$ (and/or in *glp-1* mutants) | Reference | Expression in *daf-2(e1370)* (20°C), or upon *daf-2* RNAi | Reference | *daf-16*-dependence |
|---|---|---|---|---|---|---|---|---|
| ***sod-3*** | C08A9.1 | CF1553* | Few head and tail neurons, pharynx and vulva. | Strong induction in intestinal cells upon Z2, Z3 ablation, and in *glp-1* mutants. | [1] | Strong induction in all tissues in *daf-2(e1370);* Induced in all tissues except neurons upon *daf-2* RNAi. | [2] | [1, 3, 4] |
| ***dod-8*** | C06B3.4 | BC12544# | GFP visible in head, body and tail neurons and in muscles. Larval intestine shows expression but not in adults. | Strong induction in intestinal cells upon Z2, Z3 ablation, and in *glp-1* mutants. No elevation of GFP in neurons and muscles; if anything GFP levels are reduced further. | [1]; This study | Up-regulated in *daf-2(-)* microarrays; GFP reporter does not show intestinal induction upon *daf-2* RNAi, or in *daf-2(e1370)* mutants at 20°C. Expression strongly increased in neurons and muscles. Intestinal induction seen at 25°C; *Pdod-8::RFP* with larger promoter shows intestinal induction in *daf-2(e1370)* at 20°C too. | [4]; This Study; P. Zhang and C. Kenyon, unpublished | [1, 4]; P. Zhang and C. Kenyon, unpublished |
| ***gpd-2*** | K10B3.8 | BC11128^ | Larvae and adults exhibit GFP in intestine, body wall muscles, neurons and hypodermis. | Z2, Z3 ablation causes GFP induction in intestinal cells; Recapitulated in *glp-1* mutants. | [1]; This study | Up-regulated in *daf-2*(-) microarrays; *daf-2* RNAi produces increased GFP expression in intestine; In *daf-2(e1370)* mutants GFP elevated in intestine & muscles. | [4]; P. Zhang and C. Kenyon, unpublished; This study | [1, 4] |
| ***nnt-1*** | C15H9.1 | BC10466# | GFP visible in head and tail neurons, coelomocytes, pharynx and occasionally, faintly in the intestine. | Z2, Z3 ablation causes GFP induction in neurons and intestinal cells; Recapitulated in *glp-1* mutants. | [1]; This study | Up-regulated in *daf-2*(-) microarrays; *daf-2* RNAi produces increased intestinal GFP; In *daf-2(e1370)* mutants GFP induced in intestine & hypodermis. | [4]; P. Zhang and C. Kenyon, unpublished; This study | [1, 4] |
| ***K07B1.4*** | K07B1.4 | BC14344# | Larval and adult expression clearly evident in intestinal cells and muscles. | Z2, Z3 ablation causes strong increase in adult intestine. Muscle expression rarely induced. Recapitulated in *glp-1* mutants. | This study | Up-regulated in *daf-2*(-) microarrays; *daf-2* RNAi does not elevate GFP levels; In *daf-2(e1370)* mutants muscle (and rarely intestinal) GFP elevated. | [4]; P. Zhang and C. Kenyon, unpublished; This study | [4] |
| ***T21D12.9*** | T21D12.9 | BC14516^ | Moderate intestinal expression in adults. GFP also visible in pharynx and hypodermis. | Z2, Z3 ablation causes strong increase in intestinal GFP levels. Recapitulated in *glp-1* mutants. | This study | Up-regulated in *daf-2*(-) microarrays; *daf-2* RNAi produces no change in GFP expression. | [4]; P. Zhang and C. Kenyon, unpublished | [4] |
| | | | | | | | | |

| | | | | | | | | |
|---|---|---|---|---|---|---|---|---|
| ***F52H3.5*** | F52H3.5 | BC15603^ | Larval and adult intestines exhibit low to moderate GFP levels. Also visible in pharynx and head neurons. | Z2, Z3 ablation causes strong increase in intestinal GFP, and moderate up-regulation in some head neurons and pharynx. Recapitulated in *glp-1* mutants. | This study | Up-regulated in *daf-2(-)* microarrays; *daf-2* RNAi does not show GFP up-regulation; In *daf-2(e1370)* mutants inconsistent increase in pharyngeal GFP is seen. No intestinal induction noticed. | [4]; P. Zhang and C. Kenyon, unpublished; This study | [4] |
| ***aat-1*** | F27C8.1 | BC14620# | Larval and adult expression visible in intestine, neurons and somatic gonad. | In *glp-1* mutants, GFP is elevated in intestinal cells. | This study | No change detected in mRNA levels in *daf-2* mutants. | Lee et al., 2003 | [5] |
| ***pssy-1*** | ZC506.3 | BC14795# | GFP visible in intestine, body wall muscles and neurons. | In *glp-1* mutants, expression is moderately up-regulated in intestinal cells. | This study | No change detected in mRNA levels in *daf-2* mutants. | Lee et al., 2003 | [5] |
| ***dod-16*** | B0213.15 | BC13846# | Larval and adult intestinal cells exhibit GFP. Anterior cells show higher expression. GFP visible in pharynx too. | Z2, Z3 ablation strongly increases intestinal expression. | This study | Up-regulated in *daf-2*(-) microarrays; *daf-2* RNAi does not show elevation of GFP levels. | [4]; P. Zhang and C. Kenyon, unpublished. | [4] |
| ***snt-4*** | T23H2.2 | BC11473^ | GFP visible in head and body neurons. Low expression in few intestinal cells. | Z2, Z3 ablation causes increased expression in head neurons. | This study | Up-regulated in *daf-2*(-) microarrays; *daf-2* RNAi does not show up-regulation of GFP. | [4]; P. Zhang and C. Kenyon, unpublished. | [4] |
| ***snx-1*** | C05D9.1 | BC14109# | Extensive neuronal expression. Other tissues, including the intestine, show GFP occasionally. | Z2, Z3 ablation causes elevation of GFP in head neurons. Some induction in intestinal cells noticed rarely. | This study | Up-regulated in *daf-2*(-) microarrays; *daf-2* RNAi does not show up-regulation of GFP levels. | [4]; P. Zhang and C. Kenyon, unpublished. | [4] |
| ***tps-1*** | ZK54.2 | BC14885^ | Adult expression visible in body wall muscles, head neurons and few other cells. | No change in expression upon Z2, Z3 ablation. | This study | Up-regulated in *daf-2*(-) microarrays; *daf-2* RNAi causes up-regulation of intestinal GFP; In *daf-2(e1370)* intestine & muscles GFP elevated. | [4]; P. Zhang and C. Kenyon, unpublished. | [4] |
| ***tps-2*** | F19H8.1 | BC14876^ | Adults exhibit GFP in muscle cells. | No change in expression upon Z2, Z3 ablation. | This study | Up-regulated in *daf-2*(-) microarrays; *daf-2* RNAi causes elevation of GFP in muscles & hypodermis; Expression in *daf-2(e1370)* increased in muscles & hypodermis. | [4]; P. Zhang and C. Kenyon, unpublished. | [4] |
| | | | | | | | | |

| | | | | | | | | |
|---|---|---|---|---|---|---|---|---|
| ***tnt-2*** | F53A9.10 | BC13923^ | Adults exhibit GFP in body wall muscles, other muscle cells and vulva. | No change in muscle expression upon Z2, Z3 ablation. | This study | Up-regulated in *daf-2*(-) microarrays; *daf-2* RNAi causes up-regulation of GFP in muscles. | [4]; P. Zhang and C. Kenyon, unpublished. | [4] |
| ***fat-7*** | F10D2.9 | BC15777# | Intestinal expression visible in adults. | No change in expression upon Z2, Z3 ablation. | This study | Up-regulated in *daf-2*(-) microarrays; *daf-2* RNAi causes up-regulation of GFP in intestine. | [4]; P. Zhang and C. Kenyon, unpublished. | [4] |
| ***tre-4*** | F15A2.2 | BC15383^ | Wide-spread GFP expression in adults, including intestinal cells. | No change in expression upon Z2, Z3 ablation. | This study | Up-regulated in *daf-2*(-) microarrays; *daf-2* RNAi causes up-regulation of GFP in intestine. | [4]; P. Zhang and C. Kenyon, unpublished. | [4] |

$ : Z2 and Z3 are the two germline precursors present in L1 larvae. They proliferate and give rise to the entire germline of the adult worm. Laser ablation of these cells results in loss of the germline and sterility [6].

*: Generated in the Kenyon Lab

#: Obtained from Prof. David Baillie's Lab (Strain construction supported by Genome British Columbia and Genome Canada) [7].

^: Obtained from the CGC (supported by the National Institute of Health- National Centre for Research Resources) [7].

**References:**

1. Yamawaki, TM, Arantes-Oliveira N, Berman JR, Zhang P, Kenyon C (2008) Distinct activities of the germline and somatic reproductive tissues in the regulation of *Caenorhabditis elegans'* longevity. Genetics 178: 513-526.

2. Libina, N, Berman JR, Kenyon C (2003) Tissue-specific activities of *C. elegans* DAF-16 in the regulation of lifespan. Cell 115: 489-502.

3. Honda, Y, Honda S (1999) The *daf-2* gene network for longevity regulates oxidative stress resistance and Mn-superoxide dismutase gene expression in *Caenorhabditis elegans*. Faseb J 13: 1385-1393.

4. Murphy, CT, McCarroll, SA, Bargmann, CI, Fraser, A, Kamath, RS *et al.* (2003) Genes that act downstream of DAF-16 to influence the lifespan of *Caenorhabditis elegans*. Nature 424: 277-283.

5. Lee, SS, Kennedy S, Tolonen AC, Ruvkun G (2003) DAF-16 target genes that control *C. elegans* life-span and metabolism. Science 300: 644-647.

6. Hsin, H, Kenyon C (1999) Signals from the reproductive system regulate the lifespan of *C. elegans*. Nature 399: 362-366.

7. McKay, SJ, Johnsen, R, Khattra, J, Asano, J, Baillie, DL *et al.* (2003) Gene expression profiling of cells, tissues, and developmental stages of the nematode *C. elegans*. Cold Spring Harb Symp Quant Biol 68: 159-169.
