# Supplementary material for: A Transcription Elongation Factor That Links Signals from the Reproductive System to Lifespan Extension in Caenorhabditis elegans
Source: PLoS Genet. 2009 Sep 11;5(9):e1000639. doi: 10.1371/journal.pgen.1000639 (PMC2729384; doi:10.1371/journal.pgen.1000639)
Supplement: Table S4 — Effect of daf-16(mu86) and kri-1(ok1251) mutations on lifespan extension induced by tcer-1 overexpression. (0.11 MB PDF) [file pgen.1000639.s009.pdf]

Table S4: Effect of *daf-16* and *kri-1* mutations on lifespan extension induced by *tcer-1* overexpression.

Genotype	Mean LS ± SEM (days)	Events/ Obs[a]	P vs CF2032	P vs. *daf-16(-), kri-1(-) or* N2[#]	P Line 1 vs Line 2
N2*	18.4 ± 0.3	66/98	<0.0001		
CF2032* *tcer-1 OE*	21.1 ± 0.2	79/102			
CF1037* *daf-16(mu86)*	17.5 ± 0.2	65/81	<0.0001	0.03[#]	
CF2432a *daf-16(mu86); tcer-1 OE Line #3*	17.7 ± 0.4	77/96	<0.0001	0.2	0.9
CF2432b* *daf-16(mu86); tcer-1 OE Line #9*	17.6 ± 0.2	89/104	<0.0001	0.1, 0.5[#]	
N2	16.5 ± 0.3	90/138	<0.0001		
CF2032 *tcer-1 OE*	20.5 ± 0.4	97/130			
CF1037 *daf-16(mu86)*	14.9 ± 0.2	103/135	<0.0001	<0.0001[#]	
CF2432a *daf-16(mu86); tcer-1 OE Line #3*	16.9 ± 0.7	102/131	<0.0001	0.6	0.2
CF2432b *daf-16(mu86); tcer-1 OE Line #9*	16.1 ± 0.1	90/129	<0.0001	0.02, 0.2[#]	
N2	21.1 ± 0.2	88/136	0.24		
CF2032 *tcer-1 OE*	22.4 ± 0.2	72/127			
CF1037 *daf-16(mu86)*	15.6 ± 0.3	88/128	<0.0001	<0.0001[#]	
CF2432a *daf-16(mu86); tcer-1 OE Line #3*	16.6 ± 0.2	85/135	<0.0001	0.4	0.2
CF2432b *daf-16(mu86); tcer-1 OE Line #9*	17.6 ± 0.4	89/12	<0.0001	0.001	
N2**	16.5 ± 0.3	90/138	<0.0001		
CF2032** *tcer-1 OE*	20.5 ± 0.4	97/130			

CF2052** *kri-1(ok1251)*	15.9 ± 0.2	69/135	<0.0001	0.19#	
CF2466** *kri-1(ok1251); tcer-1 OE Line #1*	16.0 ± 0.5	79/128	<0.0001	0.4, 0.5#	0.4
CF2466b *kri-1(ok1251); tcer-1 OE Line #2*	17.1 ± 0.4	74/136	<0.0001	0.6	
N2	21.1 ± 0.2	88/136	0.24		
CF2032 *tcer-1 OE*	22.4 ± 0.2	72/127			
CF2052 *kri-1(ok1251)*	16.1 ± 0.2	61/128	<0.0001	<0.0001#	
CF2466a *kri-1(ok1251); tcer-1 OE Line #1*	16.8 ± 0.3	96/130	<0.0001	0.8	0.09
CF2466b *kri-1(ok1251); tcer-1 OE Line #2*	15.9 ± 0.2	88/128	<0.0001	0.3	
N2	18.5 ± 0.1	61/96	0.0001		
CF2032 *tcer-1 OE*	21.5 ± 0.2	76/111			
CF2466a *kri-1(ok1251); tcer-1 OE Line #1*	16.6 ± 0.3	102/120	<0.0001	0.13#	0.4
CF2466a Non-transgenic control siblings	16.4 ± 0.4	91/114	<0.0001	0.03#	
CF2466b *kri-1(ok1251); tcer-1 OE Line #2*	16.9 ± 0.4	103/115	<0.0001	0.2#	0.7
CF2466b Non-transgenic control siblings	16.7 ± 0.6	94/117	<0.0001	0.1	
N2	18.5 ± 0.3	94/116	0.0005		
CF2032 *tcer-1 OE*	21.1 ± 0.2	83/116			
CF2466a *kri-1(ok1251); tcer-1 OE Line #1*	17.9 ± 0.7	106/118	0.0001	0.13#	0.9

CF2466a<br>Non-transgenic control siblings	18.3 ± 0.4	93/120	0.0008	0.03#	
CF2466b<br>*kri-1(ok1251); tcer-1 OE Line #2*	18.4 ± 0.6	91/116	0.001	0.7#	0.6
CF2466b<br>Non-transgenic control siblings	18.5 ± 0.04	93/114	<0.0001	0.8#	

LS: Lifespan; SEM: Standard error of the mean.

[a] Some animals were censored as described in Materials and Methods.

*, ** Experiments depicted in Figures 5B and 7G, respectively.
