# Supplementary material for: A Transcription Elongation Factor That Links Signals from the Reproductive System to Lifespan Extension in Caenorhabditis elegans
Source: PLoS Genet. 2009 Sep 11;5(9):e1000639. doi: 10.1371/journal.pgen.1000639 (PMC2729384; doi:10.1371/journal.pgen.1000639)
Supplement: Table S5 — Effect of tcer-1 reduction of function on the lifespans of other long-lived mutants. (0.11 MB PDF) [file pgen.1000639.s010.pdf]

Table S5: Effect of *tcer-1* reduction of function on the extended lifespans of other longevity mutants.

Genotype	RNAi treatment (if applicable)	Trial	Mean LS ± SEM (days)	Events/ Obs[a]	% of RNAi control lifespan[b]	P vs. control
daf-2(e1370)	Control	1**	45.6 ± 1.5	58/77		
	tcer-1		47.5 ± 2.0	45/63	104.1	0.12
	daf-16		16.2 ± 0.1	45/73	35.5	<0.0001
	Control	2	43.9 ± 1.9	59/90		
	tcer-1		38.3 ± 0.5	53/84	87.2	0.3
	daf-16		20.7 ± 0.5	79/80	47.1	<0.0001
daf-2(e1368)	Control	1**	31.0 ± 1.3	47/76		
	tcer-1		32.0 ± 0.8	41/75	103.2	0.54
	daf-16		19.7 ± 0.4	35/69	63.5	<0.0001
	Control	2	28.9 ± 0.6	57/91		
	tcer-1		27.4 ± 0.7	57/90	94.8	0.95
	daf-16		20.0 ± 0.2	58/90	69.2	<0.0001
daf-2(mu150)	Control	1	29.9 ± 0.7	78/105		
	tcer-1		29.3 ± 0.6	88/105	97.9	0.28
	daf-16		16.1 ± 0.4	73/105	53.8	<0.0001
eat-2(ad1116)	Control	1	26.1 ± 0.3	79/87		
	tcer-1		29.0 ± 0.5	79/88	111.1	<0.0001
	daf-16		24.6 ± 0.5	67/85	94.2	0.08
	Control	2	27.1 ± 1.1	57/91		
	tcer-1		25.7 ± 0.8	64/95	94.8	0.2
	Control	3[##]	26.5 ± 0.4	59/72		
	tcer-1		27.2 ± 0.5	40/71	102.6	0.2
isp-1(qm150)	Control	1[#]	24.1 ± 0.5	39/91		
	tcer-1		26.1 ± 0.5	52/90	108.2	0.0005
	daf-16		19.7 ± 0.5	55/90	81.7	0.05
daf-2(e1370)		1*	47.2 ± 0.7	91/103		
tcer-1(tm1452); daf-2(e1370)			46.6 ± 0.6	58/102	98.7	0.43
daf-2(e1370)		2	41.6 ± 0.5	92/120		

tcer-1(tm1452); daf-2(e1370)		42.5 ± 0.4	70/126	102.1	0.2
daf-2(e1368)	1*	33.1 ± 0.4	75/103		
tcer-1(tm1452); daf-2(e1368)		31.3 ± 1.8	57/100	94.5	0.1
N2		16.7 ± 0.1	70/113		
daf-2(e1368)	2	26.6 ± 0.7	91/132		
tcer-1(tm1452); daf-2(e1368)		23.3 ± 0.5	75/136	87.5	0.2

LS: Lifespan; SEM: Standard error of the mean.

[a] Some animals were censored as described in Materials and Methods.

[b] Control refers to worms exposed to empty vector plasmid without an RNAi insert (in RNAi experiments).

*, **, [#, ##] Experiments depicted in Figures 6A, B, F and G, respectively.
