# Supplementary material for: A Transcription Elongation Factor That Links Signals from the Reproductive System to Lifespan Extension in Caenorhabditis elegans
Source: PLoS Genet. 2009 Sep 11;5(9):e1000639. doi: 10.1371/journal.pgen.1000639 (PMC2729384; doi:10.1371/journal.pgen.1000639)
Supplement: Table S6 — Strains used in this study. (0.12 MB PDF) [file pgen.1000639.s011.pdf]

Table S6: Strains generated for, and used in this study.

No.	Strain	Genotype	Comments
1	CF2166	*tcer-1(tm1452)II Line #8*	
2	CF2167	*tcer-1(tm1452)II Line #9*	Used for all crosses
3	CF2152	*tcer-1(tm1452)II; glp-1(e2141ts)III Line #1.1-11.5*	
4	CF2153	*tcer-1(tm1452)II; glp-1(e2141ts)III Line #1.1-4.5*	
5	CF2154	*tcer-1(tm1452)II; glp-1(e2141ts)III Line #1.1-11.1*	Used to cross DAF-16-target gene reporters and *tcer-1 OE* into *tcer-1; glp-1* and *glp-1* backgrounds
6	CF2155	*tcer-1(tm1452)II; glp-1(e2141ts)III Line #14.1-12.5*	
7	CF2144	*muEx321 (Podr-1::rfp) Line I15-6-5*	75ng/µl injected into N2
8	CF2145	*muEx322 (Podr-1::rfp) Line I15-6-6*	75ng/µl injected into N2
9	CF2031	*muEx306 (Ptcer-1::tcer-1::gfp, Podr-1::rfp)*	*tcer-1:* 50ng/µl and *odr-1* 75ng/µl injected into N2
10	CF2032	*muEx307 (Ptcer-1::tcer-1::gfp, Podr-1::rfp)*	Concentrations same as CF2031; Used for all ablations, *tcer-1 OE* experiments and crosses
11	CF2033	*muEx308 (Ptcer-1::tcer-1::gfp, Podr-1::rfp)*	Same as CF2031
12	CF2034	*muEx309 (Ptcer-1::tcer-1::gfp, Podr-1::rfp)*	Same as CF2031
13	CF2148	*muEx325 (Ptcer-1::tcer-1::gfp, Podr-1::rfp) Line #I16-2-1*	*tcer-1:* 20ng/µl and *odr-1* 75ng/µl injected into N2
14	CF2149	*muEx326 (Ptcer-1::tcer-1::gfp, Podr-1::rfp) Line #I16-2-5*	--do--
15	CF2150	*muEx327 (Ptcer-1::tcer-1::gfp, Podr-1::rfp) Line #I16-1-5*	--do--
16	CF2151	*muEx328 (Ptcer-1::tcer-1::gfp, Podr-1::rfp) Line #I16-1-1*	--do--
17	CF2168	*tcer-1(tm1452)II; daf-2(e1370)III*	
18	CF2169	*tcer-1(tm1452)II; daf-2(e1378)III*	
19	CF2175	*glp-1(e2141ts)III; muEx307 (Ptcer-1::tcer-1::gfp, Podr-1::rfp) Line #32-10*	
20	CF2176	*glp-1(e2141ts)III; muEx307 (Ptcer-1::tcer-1::gfp, Podr-1::rfp) Line #32-36*	
21	CF2177	*glp-1(e2141ts)III; muEx307 (Ptcer-1::tcer-1::gfp, Podr-1::rfp) Line #32-40*	
22	CF2178	*glp-1(e2141ts)III; muEx308 (Ptcer-1::tcer-1::gfp, Podr-1::rfp) Line #33-16*	
23	CF2179	*glp-1(e2141ts)III; muEx308 (Ptcer-1::tcer-1::gfp, Podr-1::rfp) Line #33-10*	

24	CF2180	*glp-1(e2141ts)III; muEx308 (Ptcer-1::tcer-1::gfp, Podr-1::rfp) Line #33-17*	
25	CF2432a	*daf-16(mu86)I; muEx307 (Ptcer-1::tcer-1::gfp, Podr-1::rfp) Line #3*	
26	CF2432b	*daf-16(mu86)I; muEx307 (Ptcer-1::tcer-1::gfp, Podr-1::rfp) Line #9*	
27	CF2466a	*kri-1(ok1251)I; muEx307 (Ptcer-1::tcer-1::gfp, Podr-1::rfp) Line #1*	
28	CF2466b	*kri-1(ok1251)I; muEx307 (Ptcer-1::tcer-1::gfp, Podr-1::rfp) Line #2*	
29	CF2857	*tcer-1(tm1452)II; muEx307 (Ptcer-1::tcer-1::gfp, Podr-1::rfp) Line #16*	
30	CF2858	*tcer-1(tm1452)II; muEx307 (Ptcer-1::tcer-1::gfp, Podr-1::RFP) Line# 18*	
31	CF2859a	*tcer-1(tm1452)II; glp-1(e2141ts)III (Ptcer-1::tcer-1::gfp, Podr-1::rfp)<br>Line #24*	
32	CF2859b	*tcer-1(tm1452)II; glp-1(e2141ts)III (Ptcer-1::tcer-1::gfp, Podr-1::rfp)<br>Line #81*	
33	CF2859c	*tcer-1(tm1452)II; glp-1(e2141ts)III (Ptcer-1::tcer-1::gfp, Podr-1::rfp)<br>Line #91*	
34	CF2860	*daf-2(e1370)III; muEx307 (Ptcer-1::tcer-1::gfp, Podr-1::rfp) Line #10*	
35	CF3025a	*eat-2(ad1116)II muEx307 (Ptcer-1::tcer-1::gfp, Podr-1::rfp) Line #1*	
36	CF3025b	*eat-2(ad1116)II muEx307 (Ptcer-1::tcer-1::gfp, Podr-1::rfp) Line #2*	
37	CF3124	*glp-1(e2141ts)III; Pgpd-2::gfp Line# 4*	
38	CF3125a	*tcer-1(tm1452)II; glp-1(e2141ts)III; Pgpd-2::gfp Line #6*	
39	CF3197a	*glp-1(e2141ts)III; sEx14344 (rCesK07B1.4::gfp + pCeh361) Line #3*	
40	CF3198a	*tcer-1(tm1452)II; glp-1(e2141ts)III; sEx14344 (rCesK07B1.4::gfp + pCeh361) Line #36*	
41	CF3201a	*glp-1(e2141ts)III; sEx10466 (rCesC15H9.1::gfp + pCeh361) Line #28*	
42	CF3202a	*tcer-1(tm1452)II; glp-1(e2141ts)III; sEx10466 (rCesC15H9.1::gfp + pCeh361) Line #7*	
43	CF3204	*glp-1(e2141ts)III; sEx14516 (rCesT21D12.9::gfp + pCeh361)<br>Line #10*	
44	CF3229	*tcer-1(tm1452)II; glp-1(e2141ts)III; sEx14516 (rCesT21D12.9::gfp + pCeh361) Line #68*	

45	CF3250	*glp-1(e2141ts)III; sEx15603 (rCesF52H3.5::gfp + pCeh361) Line #10*	
46		*tcer-1(tm1452)II; glp-1(e2141ts)III; sEx15603 (rCesT21D12.9::gfp + pCeh361) Line #17*	
47	CF2352	*tcer-1(tm1452)II; glp-1(e2141ts)III; Pdod-8::gfp*	
48	CF3253a	*tcer-1(tm1452)II; glp-1(e2141ts)III Psod-3::gfp Line #52*	
49		*daf-16(mu86)I; tcer-1(tm1452)II; glp-1(e2141ts)III; muIs109(Pdaf-16::daf-16::gfp, Podr-1::rfp)*	
**Strains Obtained from the CGC or David Baillie's laboratory**			
50	BC11128	*dpy-5(e907)I; sEx11128 (rCesK10B3.8::gfp + pCeh361)*	
51	BC14344	*dpy-5(e907)I; sEx11128 (rCesK07B1.4::gfp + pCeh361)*	
52	BC10466	*dpy-5(e907)I; sEx10466 (rCesC15H9.1::gfp + pCeh361)*	
53	BC14516	*dpy-5(e907)I; sEx14516 (rCesT21D12.9::gfp + pCeh361)*	
54	BC15603	*dpy-5(e907)I; sEx15603 (rCesF52H3.5::gfp + pCeh361)*	
**Other strains**			
55	CF1903	*glp-1(e2141ts)III*	outcrossed thrice to Kenyon lab N2
56		*glp-1(e2141ts)III*	
57	CF1908	*eat-2(ad1116)II*	outcrossed thrice to Kenyon lab N2
58	DA1116	*eat-2(ad1116)II*	
59	CF1041	*daf-2(e1370)III*	
60	DR1572	*daf-2(e1368)III*	
61	CF1844	*fer-15(b26)II; daf-2(mu150); fem-1(hc17)IV*	
62	MQ887	*isp-1(qm150)IV*	
63	CF2050	*kri-1(ok1251)I*	
64	CF1037	*daf-16(mu86)I*	
65	CF2630	*sIs10314 (Pdod-8::gfp + PCeh361)*	BC12544 crossed to N2
66	CF2573	*glp-1(e2141ts)III; sIs10314 (Pdod-8::gfp)*	
67	CF2790	*daf-2(e1370)III; sIs10314 (Pdod-8::gfp)*	
68	CF1935	*daf-16(mu86)I; glp-1(e2141)III; muIs109 (Pdaf-16::daf-16::gfp, Podr-1::rfp)*	
69	CF1929	*glp-1(e2141ts)III; muIs84 (Psod-3::gfp)*	
