# Supplementary material for: A Transcription Elongation Factor That Links Signals from the Reproductive System to Lifespan Extension in Caenorhabditis elegans
Source: PLoS Genet. 2009 Sep 11;5(9):e1000639. doi: 10.1371/journal.pgen.1000639 (PMC2729384; doi:10.1371/journal.pgen.1000639)
Supplement: Table S7 — Q-PCR primers used in this study. (0.07 MB PDF) [file pgen.1000639.s012.pdf]

Table S7: Q-PCR primers used in this study.

Gene	Forward	Reverse
tcer-1	5’ GAT CAT ATT GAA AAC TTG GGC CGC 3’	5’ TTA TTG CTT TCT GCG ATC CCG CTC 3’
dod-8	5’ ACA GGA TGT CTT CAA AAG GAA TAT GG 3’	5’ TTG CTG GGG TGA TAG CTT GG 3’
sod-3	5’ CTA AGG ATG GTG AAC CTT CA 3’	5’ CGC GCT TAA TAG TGT CCA TCA G 3’
sod-3	5’ AAA GGA GCT GAT GGA CAC TAT TAA GC 3’	5’ AAG TTA TCC AGG GAA CCG AAG TC 3’
gpd-2	5’ AAG GCC AAC GCT CAC TTG AA 3’	5’ GGT TGA CTC CGA CGA CGA AC 3’
nnt-1	5’ CAG TAG AAA CTG CTG ACA TGC TTC 3’	5’ GAG CGA TGG GAT ATT GTG CCT GAG 3’
K07B1.4	5’ GGT CTT CTT CCA TTC AGA AAA CC 3’	5’ TGT ATG TCT GAT GAA GTG TGT CG 3’
T21D12.9	5’ CAT CTA AAT CTA TCA ACT AAT AGA G 3’	5’ GTA GGA CAG GTC CAA AAC TTC CAA G 3’
pssy-1	5’ GGA ATG ATT TCG ATT GGG GAT CC 3’	5’ TGT GTT CAG CTC GGT TAG CAA CC 3’
F52H3.5	5’ GAA GTT TAC AAA AGC ACT CGA AG 3’	5’ GGT TTA TTT TGA AGT CGG TAT GC 3’
aat-1	5’ CCC AAA ACG AAA CCT TCC ACT CGC 3’	5’ TGA AAT TGC TGT GTA GAG AGC CAC 3’
daf-16	5’ ATC CAA TTG TGC CAA GCA CTA A 3’	5’ CCA CCA TTT TGA TAG TTT CCA TAG G 3’
hsf-1	5' GAA TGC GAC TAG GCA AAT GGC 3'	5' GGT GGA TGA GGT GGA AGT CG 3'
daf-9	5' GTG ACC CAC TTG CTG GAG CG 3'	5' CGT CCA CTT GCG GAG AAT GCC 3'
daf-12	5' CGG ATT CCA AAA GCA CTG GG 3'	5' CTC CTG GCA GCT CTT CGG 3'
act-1	5’ CTA CGA ACT TCC TGA CGG ACA AG 3’	5’ CCG GCG GAC TCC ATA CC 3’
ama-1	5’ TGG AAC TCT GGA GTC ACA CC 3’	5’ CAT CCT CCT TCA TTG AAC GG 3’
csq-1	5’ AAC TGA GGT TCT GAC CGA GAA G 3’	5’ TAC TGG TCA AGC TCT GAG TCG TC 3’
pmp-3	5’ GTT CCC GTG TTC ATC ACT CAT 3’	5’ ACA CCG TCG AGA AGC TGT AGA 3’
nhr-23	5’ CAG AAA CAC TGA AGA ACG CG 3’	5’ CGA TCT GCA GTG AAT AGC TC 3’
Y45F10D.4	5’ GTC GCT TCA AAT CAG TTC AGC 3’	5’ GTT CTT GTC AAG TGA TCC GAC A 3’
